# Is Participatory Design Associated with the Effectiveness of Serious Digital Games for Healthy Lifestyle Promotion? A Meta-Analysis

**DOI:** 10.2196/jmir.4444

**Published:** 2016-04-29

**Authors:** Ann DeSmet, Debbe Thompson, Tom Baranowski, Antonio Palmeira, Maïté Verloigne, Ilse De Bourdeaudhuij

**Affiliations:** ^1^Ghent UniversityDepartment of Movement and Sport SciencesGhentBelgium; ^2^Baylor College of MedicineUnited States Department of Agriculture/Agricultural Research Service (USDA/ARS) Children’s Nutrition Research CenterHouston, TXUnited States; ^3^University of LisbonUniversidade Lusófona de Humanidades e TecnologiasFaculty of Physical Education and Sports and Interdisciplinary Centre for the Study of Human Performance (CIPER) – Faculty of Human MovementLisbonPortugal

**Keywords:** serious games, video games, computer games, games, health promotion, meta-analysis, review, design, community-based participatory research

## Abstract

**Background:**

Serious digital games can be effective at changing healthy lifestyles, but large differences in their effectiveness exist. The extent of user involvement in game design may contribute to game effectiveness by creating a better fit with user preferences. Participatory design (PD), which represents active user involvement as informant (ie, users are asked for input and feedback) or codesigner (ie, users as equal partners in the design) early on and throughout the game development, may be associated with higher game effectiveness, as opposed to no user involvement or limited user involvement.

**Objective:**

This paper reports the results of a meta-analysis examining the moderating role of PD in the effectiveness of serious digital games for healthy lifestyle promotion.

**Methods:**

Four databases were searched for peer-reviewed papers in English that were published or in press before October 2014, using a (group-) randomized controlled trial design. Effectiveness data were derived from another meta-analysis assessing the role of behavior change techniques and game features in serious game effectiveness.

**Results:**

A total of 58 games evaluated in 61 studies were included. As previously reported, serious digital games had positive effects on healthy lifestyles and their determinants. Unexpectedly, PD (g=0.075, 95% CI 0.017 to 0.133) throughout game development was related to lower game effectiveness on behavior (Q=6.74, *P*<.05) than when users were only involved as testers (g=0.520, 95% CI 0.150 to 0.890, *P*<.01). Games developed with PD (g=0.171, 95% CI 0.061 to 0.281, *P*<.01) were also related to lower game effectiveness on self-efficacy (Q=7.83, *P*<.05) than when users were not involved in game design (g=0.384, 95% CI 0.283 to 0.485, *P*<.001). Some differences were noted depending on age group, publication year of the study, and on the specific role in PD (ie, informant or codesigner), and depending on the game design element. Games developed with PD were more effective in changing behavioral determinants when they included users in design elements on game dynamics (beta=.215, 95% CI .075 to .356, *P*<.01) and, more specifically, as an informant (beta=.235, 95% CI .079 to .329, *P*<.01). Involving users as informants in PD to create game levels was also related to higher game effectiveness (Q=7.02, *P*<.01). Codesign was related to higher effectiveness when used to create the game challenge (Q=11.23, *P*<.01), but to lower game effectiveness when used to create characters (Q=4.36, *P*<.05) and the game world (Q=3.99, *P*<.05).

**Conclusions:**

The findings do not support higher effectiveness of games developed with PD. However, significant differences existed among PD games. More support was found for informant roles than for codesign roles. When PD was applied to game dynamics, levels, and game challenge, this was associated with higher effectiveness than when it was applied to game aesthetics. Since user involvement may have an important influence on reach, adoption, and implementation of the intervention, further research and design efforts are needed to enhance effectiveness of serious games developed with PD.

## Introduction

Serious digital games are a form of organized play, using a digital device, intended to be both entertaining and educational [1]. Serious games have shown promising effects in promoting healthy lifestyles [2,3]. Healthy lifestyles can prevent a wide range of diseases, such as some cancers, cardiovascular diseases, stroke, dementia, mental illness, and diabetes [4-7], and having effective interventions to promote these lifestyles is therefore of great public health importance. A recent meta-analysis of serious games for healthy lifestyle promotion revealed that serious games were effective at changing (1) behavior (eg, number of steps taken per day), (2) individual determinants of this behavior (eg, knowledge, attitudes, social norms, self-efficacy, skills, and perceived environmental barriers or facilitators), and (3) clinical outcomes (eg, body mass index). Although the effects were small, they were in line with the magnitude of effects found for other computer-delivered interventions [8,9]. These effects applied across health behaviors (eg, physical activity, illness self-management, and social behavior). Games were also equally effective for both men and women and for all age groups [2]. Apart from being effective, serious games are also well liked by their target group: adolescent users preferred serious games to traditional educational approaches, such as classroom teaching [10]. Being enjoyable, absorbing, and intrinsically motivating [1,11], serious games may overcome motivational barriers that health promotion programs often encounter [12-14].

Despite their potential, large variations exist in serious game effectiveness that are not well understood [15]. The recent meta-analysis of serious games for healthy lifestyle promotion, which showed games were effective, also investigated the role of theory, individual tailoring, and sample and study characteristics in game effectiveness [2]. A game was considered effective if it reached its goal of improving healthy lifestyles (eg, being more physically active), improving the health outcomes related to these lifestyles (eg, reducing obesity), or improving determinants predictive of a healthy lifestyle (eg, having a positive attitude toward physical activity). Randomized controlled trials were included in the meta-analysis. To be deemed effective, the gain in these health-related outcomes had to be higher in the condition receiving the intervention (ie, the game) than in the control condition. Independent variables included theory, individual tailoring, study quality, health behavior, and study and sample characteristics. Theoretical foundation reflected the theory used to guide intervention development (eg, behavior change theories and game-based learning theories) and was a significant moderator of game effectiveness. Individual tailoring reflected the degree to which the intervention content or appearance was adjusted to match the individual user’s characteristics (eg, girls would receive a different game challenge than boys) and was also a significant moderator of effectiveness. Of the investigated study characteristics (eg, average time during which they were exposed to the game), only the time duration between the end of the intervention and the measurement of effects affected game effectiveness.

Other characteristics, such as specific type of health behavior (eg, physical activity, preventive behavior and illness-self-management, and mental health promotion and social behavior) and study quality (ie, study validity and reliability, such as sampling method, measures, and blinding) did not affect game effectiveness.

Heterogeneity in effectiveness, however, remained that could not be fully explained by these moderators, pointing to the need to explore other potential moderators of serious game effectiveness. The extent to which serious games were developed in participatory design (PD) between users and professionals may be an important moderator, as appreciation of game features may differ by target group [16-18]. This may be particularly important when professionals do not share the same characteristics as the target group [19]. Involving end users in design should increase game fit with user preferences, which has been hypothesized to enhance game effectiveness [20].

Participatory design is a broad term that comprises several purposes, methods, and intensities of user involvement. An overarching PD principle is that users should be involved in the development of tools designed to benefit their quality of life [21]. In a pragmatic perspective, PD is considered beneficial via its potential to improve program effectiveness and user adoption [22]; our study reflects this perspective. Other perspectives on user participation consider PD to be useful regardless of its potential contribution to program effectiveness. PD is also regarded as a moral imperative motivated by its potential effects on community empowerment, giving communities (ie, groups of people with shared interests, needs, or identities) more control over their lives by active involvement in program design [22].

PD has similarities to other concepts of user involvement in game design. It is closely related to the concept of user-centered design (UCD), which emphasizes continuous user involvement at every design stage with iterative testing. UCD considers PD to be one of its approaches [23]. Formative research is also frequently mentioned as a way to involve users in health game development [24]. Formative research generally refers to collecting data among target users to ensure the intervention is acceptable and appropriate for them, for example, at a cultural or cognitive level [25]. This type of formative research resembles the UCD-specified role for the user as informant. However, power imbalances between users and professionals may be maintained in formative research [26]. Formative evaluation may be one method used within PD, but does not necessarily constitute PD [27].

In this paper, *user involvement* is an umbrella term describing the degree to which the end user influences game design. User involvement can take several forms: users, testers, informants, and codesign partners [28]. As users or testers, the target group is observed during game play. They are asked for acceptability and/or usability of an early version of the game developed without user input—also known as alpha testing [29,30]. These forms of user involvement are not considered PD, since the experts initiated the design and made the final decisions without user involvement. In the mode of informant—users are asked for input and feedback—or codesigner—users as equal partners in the design—here considered forms of PD, users are actively involved and are asked for input starting at an early stage of design, prior to product development [28].

Active user involvement in the development of health promotion interventions has been advocated to ensure user concerns are adequately reflected in the program [31], to integrate user and professional expertise for mutual learning [32], and to increase community acceptability and adoption of the intervention [19,32]. The benefits of user involvement on program effectiveness are rarely assessed [33]. Collecting hard evidence on the value of PD is difficult, as its fuzzy processes are considered irreconcilable with commonly used research methods for outcome measurements [22]. An experimental design with and without user participation may not be practically feasible, since insights into which factors to control in a comparison condition may surface only during the PD process [22]. Funding bodies, nevertheless, often require evidence of the contribution that PD has made to effectiveness [19,22].

A meta-analysis may advance our understanding of how PD relates to game effectiveness by quantifying and comparing differences across studies and by overcoming small sample sizes in individual studies [34]. This can add to the limited evidence of the value of PD in serious game design and effectiveness.

Several game design elements (eg, levels, challenge, feedback, and tailoring) may contribute to game effectiveness [12,35]. PD in educational games showed that users were focused on design elements, such as narrative, sounds, setting, and characters, but struggled with integrating other design elements in gameplay, such as educational content [36]. Since a recent meta-analysis on serious games indicated that only certain design elements (eg, levels and adaptive game challenge) were associated with game effectiveness (data available upon request from the authors), we could expect user involvement in these design elements to be more strongly associated with game effectiveness.

This study conducted a meta-analysis and meta-regression analysis to investigate the role of user involvement and PD in the effectiveness of serious games for healthy lifestyle promotion. The research questions included the following: (1) Does game effectiveness differ by user involvement (ie, no involvement, tester only, or PD)?, (2) Does the influence of user involvement on effectiveness differ by age group (ie, average participant age <18 years or ≥18 years) or by publication year of the study (ie, game studies published before 2010 or between 2010 and 2014)?, (3) Does PD relate differentially to game effectiveness depending on the design role for users (ie, informant or codesigner)?, and (4) Does PD relate differentially to game effectiveness depending on the design element (eg, educational content, game challenge, or narrative) in which it was applied?

## Methods

### Overview

The research protocol was inspired by Cochrane guidelines [37]. Meta-analysis reporting was conducted in accordance with the Preferred Reporting Items for Systematic Reviews and Meta-Analyses (PRISMA) statement [38], with the exception of sensitivity analyses and effectiveness data per study, which are reported elsewhere. Effectiveness data for this study were obtained from a meta-analysis evaluating the role of game design elements in serious game effectiveness. This meta-analysis was an update—search was updated for papers between 2013 and 2014—of a previous meta-analysis. For background information on effectiveness calculations, we refer the readers to an earlier meta-analysis by DeSmet et al [2].

### Search Strategy and Study Selection

#### Inclusion and Exclusion Criteria

The following were the inclusion and exclusion criteria for the studies:

1. Studies were included if they investigated serious digital games, defined as organized play with a set of rules by which to play and a goal, which created a challenge, provided feedback or showed outcomes, entailed interaction, and had a topic [1]. Studies were excluded if they investigated commercial off-the-shelf games, multimedia programs with no interaction (eg, only watching a video without a challenge), and board games.

2. Studies were included if they were designed for healthy lifestyle promotion aiming to improve health behaviors, such as healthy diet (eg, fruit and vegetable consumption), physical activity (eg, number of steps taken per day), social behavior (eg, emotion recognition and not bullying others), health responsibility and maintenance (eg, illness self-management and not smoking), and stress management or self-actualization (eg, personal growth and mental health promotion) [39]. Studies were excluded if they investigated games that only targeted an increased skill level but did not target lifestyle change (eg, athletic performance), that were only used in a therapeutic context and with no intent to create a lifestyle change (eg, treatment support), or that were used for professional education (eg, teaching medical skills).

3. Studies were included if they reported outcomes on behavior or its determinants (eg, knowledge and attitudes). Studies were excluded if they only consisted of usability evaluations, player experiences, or case studies or that only reported effects on clinical outcomes, not healthy lifestyles.

4. Studies were included if they provided data that allowed the computation of effect sizes. Studies were excluded if there were no data available in the article or after consulting authors allowing an effect size to be calculated.

5. Studies were included if they used a research design with a control condition to which either individuals or groups of individuals were randomly assigned. Studies were excluded if they consisted of a one-group, pretest post-test design or a one-group, post-test-only design.

6. Studies were included if they were reported in English.

#### Search Strategy

Four databases were searched for peer-reviewed publications since the creation of the journal databases until October 2014: PubMed (1966), Web of Science (1926), Cumulative Index to Nursing and Allied Health Literature (CINAHL) (1937), and PsycINFO (1887). The search was conducted using the keywords *games*, *video games*, or *interactive multimedia* and *health*. Search results were complemented with hand-searching studies reported in reviews, examining the table of contents of relevant specialized journals and databases (ie, Computers in Human Behavior, CyberPsychology, Behavior and Social Networking, Games for Health Journal, JMIR Serious Games, Telemedicine and E-Health, and Health Games Research database), and by requesting qualifying manuscripts from the local Digital Games Research Association (DiGRA) chapter. Authors were contacted for more information when data for coding or effect size calculation were lacking. When unclear, the coding frame was presented to the authors for completion or review and correction.

### Coding Frame

#### Primary and Secondary Outcomes

The following primary outcomes were studied: behavior and behavioral determinants (ie, knowledge, attitudes, subjective norm, perceived barriers, skills, self-efficacy, and behavioral intention). These behavioral determinants are considered the key changeable influences on behaviors [40]. Whatever the authors identified as attitudes, skills, etc, were counted in those categories. As secondary outcomes, clinical effects (eg, weight and depression score) were included, when applicable. These outcomes could relate to several healthy lifestyles, in accordance with the Health-Promoting Lifestyle Profile scale [39]: healthy diet, physical activity, social skills and behavior, health responsibility and maintenance, and stress management and self-actualization.

#### User Preferences

A coding frame for PD of serious games was based on the items listed in Textbox 1 [12,28,41-43].

To provide an index of PD for mechanics, dynamics, and aesthetics, the number of aspects in which users were involved as informant or as codesigner were summed per area. User involvement could relate to the choice to include certain features and/or to how these features were designed. The user group was defined as the end users for whom the intervention was intended, not as the stakeholders who were important in facilitating the implementation of the intervention (eg, teachers and health professionals). Stakeholder involvement in PD was coded separately.

Also coded were the number of consultations with the users (ie, exact number or constant involvement) and stakeholders (ie, exact number or constant involvement), the method used to involve users (eg, focus groups), and the sampling strategy to recruit the user and stakeholder groups (see Multimedia Appendix 1). These process measures may indicate reliability and validity of the findings from consulting the target group [19]. The full coding frame can be found in Multimedia Appendix 1.

Two coders (ADS, Wendy Van Lippevelde) independently coded user involvement for a random selection of one-third of the game studies, showing good coder agreement (κ=.83). All authors of the included PD studies were offered an opportunity to review and, if necessary, adjust the more detailed coding sheet for their study to guarantee coding accuracy.

**Coding frame for participatory design upon which serious games were based.**

A coding frame for PD of serious games was based on the following:

1. The degree of user involvement. Users were either (1) not involved, (2) involved as testers only (of a finalized version), or (3) involved in PD, as either informants at an early stage (ie, preferences and suggestions are elicited prior to and during development) or as codesigners who were part of a design team and in charge of one or more parts of game development [28].
2. When users were involved in PD as informants or codesigners, we coded the following aspects:
  - The design elements in which they were involved, inspired by the Mechanics, Dynamics, Aesthetics (MDA) framework [41].
    1. Mechanics included controls (eg, push buttons and mouse) and actions or interactivity (eg, jump and run).
    2. Dynamics included rewards (eg, points and badges) and levels (eg, number of levels and how to level up or down).
    3. Aesthetics included narrative (eg, story), challenge (eg, game type), character looks (eg, clothing style), game world looks (eg, colors and setting), and language (eg, jargon and phrases).
  - The involvement of users in choosing behavioral change strategies employed in serious games (eg, individual tailoring [12] and feedback [42,43]).
  - The involvement of users as informants or codesigners of educational content.

### Meta-Analytic Procedure

Game effectiveness was defined as an improvement in healthy lifestyles, behavioral determinants, or clinical outcomes associated with these lifestyles. Reported effects on *behavior* reflect average effects on the entire category of healthy lifestyles (eg, physical activity and healthy diet). Reported effects on *behavioral determinants* also show average effects on all behavioral determinants considered as one category (eg, knowledge and attitudes). Similarly, all clinical outcomes are considered as one category across health areas (eg, body mass index and depression scores). Hedges’ g was calculated as the effect size index, which corrects for small sample sizes [44]. A positive or a negative Hedges’ g indicated that the serious game respectively increased or reduced adoption of a healthy lifestyle or its determinants. In cases where the intervention targeted a reduction of unhealthy lifestyles, the computed sign of the effect size was reversed so all positive differences reflected an improvement in healthy lifestyles for the treatment group compared to the control group.

Study results were pooled using a random effects model. Tests for significance of the effect size are indicated in Table 1 by the mention "within category." Two *P* values per row are reported: one for Hedges' g effect sizes (considered significant at *P*<.05) and one for Cochran’s Q values, testing for heterogeneity among studies in this category (considered significant at *P*<.05). A significant *P* value for Hedges' g indicated a significant effect of the games in this category, whereas a significant *P* value for Cochran’s Q indicated that large variations existed between these studies’ effectiveness.

Moderator analyses were conducted to explain differences in effect sizes between studies. For all moderator analyses, a mixed-effects model was used, and Cochran’s Q test and I^2^[45] were reported to investigate the degree of heterogeneity in effect sizes. Moderator analyses test whether heterogeneity can be explained by differences between several categories on the moderating variable, indicated in Table 1 by the mention "between categories." A significant *P* value associated with the Q test indicated the moderator was able to explain some heterogeneity between the studies’ effectiveness. Moderator analyses were only conducted with the categories that contained a sufficient sample. A sufficient sample was defined as having at least three studies in a category, or a minimum sample size of 250 participants per category. A sample size smaller than this was shown not to have sufficient power to detect even large, homogeneous moderator effects [46]. This minimum sample size was thus chosen to make meaningful moderator analyses, without being overly restrictive. It should be emphasized, however, that this sample size may still be underpowered to detect small or heterogeneous moderator effects, and not finding significant moderator effects should hence not be interpreted as evidence for no effect [46]. When a category did not have a sufficient sample, it was excluded from the moderator analysis, which was then performed only on the remaining categories where possible—two or more categories. When a moderator was not relevant for a study (eg, narratives are not used, as they do not fit within certain game types), the study was not included in that particular moderator analysis.

Meta-regression (ie, method-of-moments procedure) was performed for continuous moderators [47], where the slope (beta) and its *P* value indicated the importance of this moderator in understanding linear changes in effect sizes. To maintain the independence of the data, whenever necessary, effect sizes were averaged across different outcomes. All analyses were performed with Comprehensive Meta-Analysis software, version 3 (Biostat Inc, Englewood, NJ, USA). Effect sizes of 0.20-0.49 were considered small effects, 0.50-0.79 were considered moderate, and ≥0.80 were considered large [48]. The research protocol for this study is available in Multimedia Appendix 2.

## Results

### Overview

**Figure 1:**
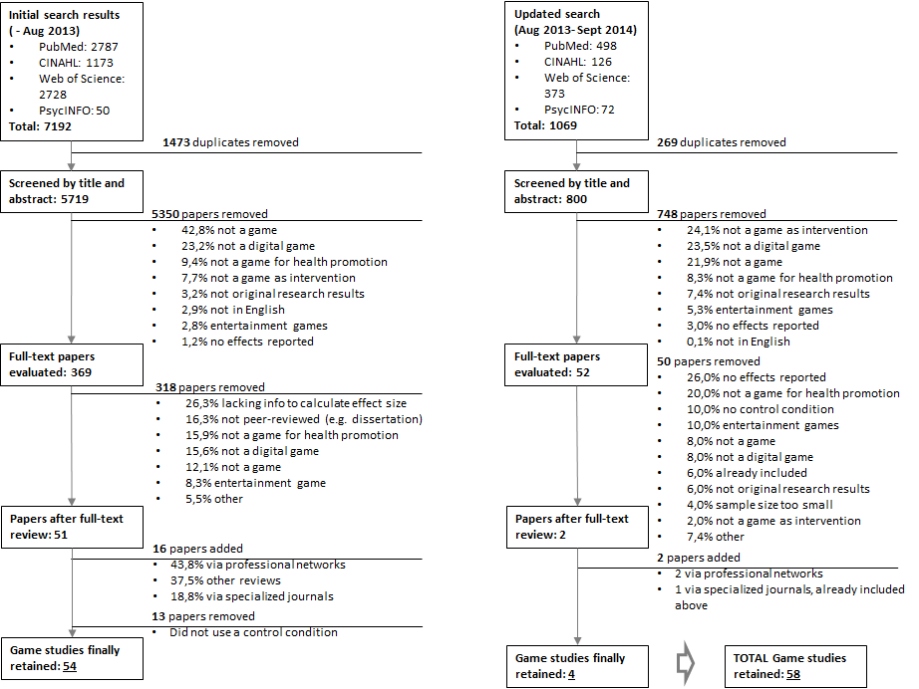
Flowchart of game study selection.

The database search was conducted at two time points: once for papers published or accepted until July 2013, and once updated for papers published or accepted until October 2014 (see Figure 1). In total, the search yielded 8261 hits, from which 1742 (21.09%) duplicates were removed. Next, 6098 articles out of 6519 (93.54%) were deleted after reading the abstract and title. After reading the full texts of the remaining articles (n=421) and adding studies from other sources (n=18), such as a search in specialized journals and databases and requests via professional networks, 58 out of 439 (13.2%) game papers were retained that fit our inclusion/exclusion criteria.

### Descriptive Information

A total of 58 games were studied in a total of 61 evaluations—3 papers studied more than one game and 3 games were also evaluated in more than one paper. Most studies came from North America (43/61, 71%), with the remainder originating from Europe (16/61, 26%) and Asia Pacific (2/61, 3%), including Australia and New Zealand. None came from Africa or Central or South America, possibly related to the English language restriction in our inclusion criteria. Most evaluated games for children (32/61, 53%) or adolescents (30/61, 49%). Around a third (21/61, 34%) evaluated games for adults, whereas only one game targeted the elderly. Over half the game studies were published in 2010 or later (33/61, 54%).

### Moderating Role of User Involvement—None, Tester, or Participatory Design—in Game Effectiveness

For 8 out of 61 studies (13%), no information on target group involvement could be obtained. Of the remaining 53 evaluations, more than half of the game evaluations involved the target group in the game design (36/53, 68%). Of these 36 studies involving the target group, 11 (31%) game evaluations only pilot-tested a finished version, and 25 out of 36 (69%) involved the target group in PD. As previously reported, serious game studies were effective in changing healthy lifestyle behavior, behavioral determinants, and clinical outcomes [2].

Table 1 lists game study effect sizes and the role of user involvement as a moderator in explaining differences between these effect sizes. Results were organized by type of outcome: behavior, behavioral determinants, and clinical outcomes. Table 1 contains several analyses per outcome. The *Total* row reports the effect sizes for all included game studies that measured effects on a particular outcome. The row, *User involvement (between categories)*, shows the results of a moderator analysis testing for differences in effect sizes between game studies of different categories of user involvement (eg, for behavior, user involvement was a significant moderator in explaining heterogeneity between game studies [Q=6.74, *P*=.03]). The ensuing rows show the average effect sizes of game studies within each category of user involvement (eg, for game studies where users were not involved, effects on behavior were not significant [g=0.540, *P*=.17], but large differences existed in effect sizes of game studies belonging to this category [Q=324.39, *P*<.001]).

**Table 1:** User involvement as moderator in game study effectiveness.

Outcome			Sample size^a^, n	k^b^, n	Hedges’ g^c^ (95% CI)	*P*_g_	Q^d^	*P*_Q_	I^2e^, %
**Behavior**									
	Total^f^		317,582	24	0.216 (0.113 to 0.319)	<.001	408.37	<.001	94
	User involvement (between categories)		11,684	22			6.74	.03	
	**User involvement (within categories)**								
		No involvement	3238	4	0.540 (-0.230 to 1.311)	.17	324.29	<.001	99
		Involvement only as tester	430	4	0.520 (0.150 to 0.890)	.01	10.21	.02	71
		Involvement as informant or codesigner	8016	14	0.075 (0.017 to 0.133)	.01	14.67	.33	11
**Behavioral determinants**									
	Total^f^		22,366	51	0. 317 (0.244 to 0.391)	<.001	219.72	<.001	77
	User involvement (between categories)		13,000	44			3.24	.20	
	**User involvement (within categories)**								
		No involvement	4649	16	0.288 (0.161 to 0.414)	<.001	39.26	.001	62
		Involvement only as tester	1335	8	0.420 (0.278 to 0.562)	<.001	9.97	.19	30
		Involvement as informant or codesigner	7016	20	0.265 (0.170 to 0.361)	<.001	49.37	<.001	62
**Self-efficacy**									
	Total^f^		14,564	21	0.227 (0.130 to 0.324)	<.001	86.14	<.001	77
	User involvement (between categories)		6398	20			7.83	.02	
	**User involvement (within categories)**								
		No involvement	1545	8	0.384 (0.283 to 0.485)	<.001	2.73	.91	0
		Involvement only as tester	434	3	0.305 (-0.044 to 0.654)	.09	6.80	.03	71
		Involvement as informant or codesigner	4419	9	0.171 (0.061 to 0.281)	.002	22.60	.004	65
**Clinical outcomes**									
	Total^f^		9488	11	0. 071 (0.031 to 0.111)	.001	8.08	.62	0
	User involvement (between categories)		1084	7			0.05	.82	
	**User involvement (within categories)**								
		No involvement	119	2	-0.036 (-0.402 to 0.330)	.85	0.20	.66	0
		Involvement only as tester	384	3	0.034 (-0.168 to 0.236)	.74	0.63	.73	0
		Involvement as informant or codesigner	700	4	-0.001 (-0.217 to 0.216]	.99	5.63	.13	47

^a^Combined participant sample size.

^b^k: number of studies.

^c^Hedges’ g (random effects).

^d^Cochran’s Q test: homogeneity statistic (mixed effects).

^e^I^2^index: inconsistency, a second measure of heterogeneity.

^f^The total includes the studies with missing values on the user involvement variable. For measures on behavior, one very large study (n=297,737) was included here, which was not involved in moderator analyses on user involvement, due to missing information.

There were significant differences (Q=6.74, *P*=.03) between the game studies’ effects on behavior depending on the nature of target group involvement. Game studies for which the target group was involved in PD (g=0.075, *P*=.01) were significantly less effective in changing behavior than game studies that were only pilot-tested among the target group after design (g=0.520, *P*=.01). Effects of game studies developed in PD with the target group showed no significant differences with game studies that had not involved the target group (g=0.540, *P*=.17) (see Table 1). We assessed whether this applied to both older (ie, published before 2010) and more recent (ie, published between 2010 and 2014) game studies. When analyzing older and recent game studies separately, the difference in effectiveness on behavior by levels of active user involvement was only significant for recent game studies (Q=3.87, *P*=.049), but not for older game studies (Q=1.15, *P*=.28). As in the full sample analysis, games where users were involved merely as testers were more effective (g=0.577, *P*=.03) than games where users were involved in PD (g=0.066, *P*=.09). There were insufficient observations available here for comparison with studies without user involvement (see Multimedia Appendix 3, Tables A3-1 and A3-2).

We also assessed whether this applied both to game studies evaluated among a younger audience (ie, average age of participants <18 years) and among an adult audience (ie, average age of participants ≥18 years). This difference was only significant in studies with, on average, adult participants (Q=4.39, *P*=.04). Game studies among adult participants where the target group had only been involved as testers were more effective (g=0.577, *P*=.03) than studies developed in PD (g=0.009, *P*=.92). There were insufficient observations available here for comparison with studies without user involvement (see Multimedia Appendix 3, Tables A3-3 and A3-4).

There were no significant differences between the effects on behavioral determinants. When examining determinants separately, user involvement only significantly moderated effects on self-efficacy (Q=7.83, *P*=.02). Game studies for which the target group had been involved in PD (g=0.171, *P*=.002) were less effective in increasing self-efficacy than game studies for which the target group had not been involved in the design (g=0.384, *P*<.001), but showed no significant differences with game studies where the target group had been involved in pilot testing (g=0.305, *P*=.09) (see Table 1). When examining this by the publication year of the studies, this difference only appeared significant (Q=21.14, *P*=.01) for recent game studies (ie, published between 2010 and 2014) and not for studies published prior to 2010 (Q=2.63, *P*=.11). Recent game studies (ie, published 2010-2014) for which the target group had been involved in PD (g=0.098, *P*=.17) were less effective in increasing self-efficacy than game studies for which the target group had only been involved as testers (g=0.483, *P*<.001), but showed no significant difference with game studies where users were not involved in the design (g=0.281, *P*=.04) (see Multimedia Appendix 3, Tables A3-1 and A3-2).

We also assessed whether this applied both to game studies evaluated among a younger audience (ie, average age of participants <18 years) and among an adult audience (ie, average age of participants ≥18 years). This difference was only significant in studies with, on average, younger participants (Q=9.66, *P*=.002). Game studies among children and adolescents where the target group had not been involved were more effective (g=0.377, *P*<.001) than studies in this age group developed in PD (g=0.127, *P*=.03). There were insufficient observations available here for comparison with studies where users were only involved as testers (see Multimedia Appendix 3, Tables A3-3 and A3-4). Differences in effects on clinical outcomes could only be assessed between two levels of involvement. This difference in effects between levels of user involvement was not significant (see Table 1).

In summary, PD was associated with less health behavior change than when users only pilot-tested a finalized game version—all game studies and those with average sample age ≥18 years. There were no significant differences in game studies where users had not been involved in the design process. Games developed in PD also related to lower effects on self-efficacy than when users were not involved in any phase of the game design—all game studies and those with average sample age <18 years—or when users were only involved in pilot testing—recent game studies.

### Differential Moderating Role of Participatory Design in Game Study Effectiveness by Design Elements

#### Descriptive Information

A total of 25 out of 36 (69%) game studies involved users in PD, either as informants or codesigners, and were available for a more in-depth exploration of the role of PD in game effectiveness. For 3 studies reported in 2 papers [49,50], no detailed information could be obtained from the authors; therefore, they were not included in the moderator analyses (see Table 2). This resulted in a total of 22 game studies for which detailed information was available.

Focus groups were the most frequently used method to consult users in the serious game development (14/22, 64%) [51-64]. A range of 2-16 focus groups informed game design. Out of 22 studies, 4 (18%) involved users via interviews [65-68], 2 (9%) via classroom discussions [58,69], and 1 (5%) via a survey [70]. In 3 studies out of 22 (14%), a user technical advisory board was established [55,57,62], and in 3 studies (14%) reported in 2 papers, target group users were recruited as members of the design team [70,71].

On average, 97.17 (SD 127.72) users per study were involved in PD of the game studies. Users were recruited via purposive sampling in 12 out of 22 studies (55%) [51-54,58,59,61,65,68,69,71], via a self-selected convenience sample (eg, flyers) in 8 studies out of 22 (36%) [55-57,60,62,63,66,70], and/or via snowball sampling in 4 out of 22 studies (18%) [55,57,62,64]. For one study, no information could be obtained on sampling method [67]. For some studies, the number of stages in which users were involved was unclear. A total of 6 out of 22 studies (27%)—discussed in 5 papers—mentioned continuous user involvement [59,70,71] or involvement in several stages [62,64]. For other studies, there was a range of 2-20 consultations, but it was unclear if these related to different design stages. Other stakeholders (eg, health professionals, parents, and teachers) were consulted in 13 out of 22 game studies (59%) [52,53,55-64,68]; on average, 24.91 (SD 22.24) other stakeholders were consulted.

**Table 2:** User involvement in game design elements.

Study^a^	N^b^	Ch^c^	CLV^d^	GL^e^	LW^f^	R^g^	L^h^	Co^i^	IA^j^	F^k^	T^l^	LC^m^
Campbell et al, 1999 [65]												I^n^
Yawn et al, 2000 [64]	C^o^	C	I	I				I	I			I
Baranowski et al, 2003 [69]		I	I	I	I	I						I
Campbell et al, 2004 [66]					I				I			I
Jago et al, 2006 [53]			I			I						I
Kato et al, 2008 [68]			I	I								I
Trepka et al, 2008 [63]			I	I								I
Thompson et al, 2009 [61]			I			I						I
Tortolero et al, 2010 [62]												I
Baranowski et al, 2011 [51]	I	I	I				I					
Dias and Aganti, 2011 [67]			I, C									
Sapouna et al, 2011 [58]	I, C	I, C	I	I	I, C			I, C	C			C
Swartz et al, 2011 [60]												I
Markham et al, 2012 [55]												I
Molnar and Kostkova, 2012 [56]		I										
Brown et al, 2012 [52]	I		I, C									
Schotland and Littman, 2012 [59]		I, C	I			I	I					
Christensen et al, 2013 [70]	I, C		I, C	I, C	I, C				I			
Majumdar et al, 2013 [54]	I	I	I			I		I	I			I
Song et al, 2013 (a-b^p^) [71]	C	C		C	C	C	I, C			C	C	C
Peskin et al, 2014 [57]												I

^a^Studies are chronologically organized by publication year.

^b^N: narrative.

^c^Ch: challenge.

^d^CLV: character looks/voice.

^e^GL: game world looks.

^f^LW: language, wording.

^g^R: rewards.

^h^L: levels.

^i^Co: controls.

^j^IA: interactivity/action.

^k^F: feedback.

^l^T: tailoring.

^m^LC: learning content.

^n^I: user involvement as informant.

^o^C: user involvement as codesigner.

^p^Two different types of games (a-b) were evaluated in this paper.

#### Moderating Role of Involvement as Informant or Codesigner in Game Study Effectiveness

As these analyses only related to some of the studies mentioned above (ie, 25 studies using PD), too few observations were available to analyze several of the moderators. This was the case for the moderating role of PD in game study effectiveness on clinical outcomes and for the moderating role of PD in game effectiveness on specific behavioral determinants (eg, attitudes and self-efficacy). Detailed analyses to assess the effectiveness of PD characteristics among PD studies by groups of publication year or average sample age were also not conducted due to insufficient observations.

Moderator analyses for PD as one category, considering informant and codesign together, are described in the following text. Results from detailed moderator analyses for the role as informants or as codesigner separately are summarized in the following text and described in detail in Multimedia Appendix 3 (Table A3-5).

The total number of aspects in which users were involved as informant or as codesigner was summed. There was no significant relationship between the number of areas in which users were involved as informants or codesigner and effectiveness on behavior (beta=.002, 95% CI -.026 to .029, *P*=.91, k=14, n=8016), nor on behavioral determinants (beta=.045, 95% CI -.009 to .098, *P*=.10, k=16, n=6155). Similar results were noted when analyzing the relationship between PD in these areas with effectiveness, separately for involvement as informant or as codesigner (see Multimedia Appendix 3, Table A3-5).

#### Moderating Role of Involvement as Informant or Codesigner in Mechanics, Dynamics, Aesthetics, or Educational Content in Game Study Effectiveness

There were insufficient studies (k=1) available to perform moderator analyses where users were involved in feedback or tailoring decisions.

##### Summed Mechanics, Dynamics, Aesthetics Areas

There were no significant relationships between degree of PD in mechanics (beta=-.030, 95% CI -.117 to .056, *P*=.20, k=14, n=8016), dynamic aspects (beta=.074, 95% CI -.040 to .187, *P*=.20, k=14, n=8016), or aesthetic aspects (beta=-.001, 95% CI -.038 to .035, *P*=.94, k=14, n=8016) and effectiveness on behavior.

There were also no significant relationships between degree of PD in mechanics (beta=-.078, 95% CI -.343 to .188, *P*=.57, k=16, n=6155) or aesthetic aspects (beta=.046, 95% CI -.030 to .121, *P*=.24, k=16, n=6155) and effectiveness on behavioral determinants. There was, however, a positive, significant relationship between the degree of PD in dynamics and effectiveness on behavioral determinants (beta=.215, 95% CI .075 to .356, *P*=.003, k=16, n=6155). Similar results were noted when analyzing the relationship between PD in these areas with effectiveness, separately for involvement as informant (beta=.235, 95% CI .079 to .329, *P*=.003, k=16, n=6155), but not for involvement as codesigner (see Multimedia Appendix 3, Table A3-5).

##### Specific Mechanics, Dynamics, Aesthetics Areas

We examined the role of PD as informant or codesigner in specific MDA areas of game design in explaining game study effectiveness. Significance of moderators is summarized in Table 3. Full information on these moderator analyses is provided in Multimedia Appendix 3 (Tables A3-7 to A3-15).

**Table 3:** Overview of significance of moderator analyses for the role of participatory design in specific Mechanics, Dynamics, Aesthetics design elements in game study effectiveness.

Comparisons between categories of involvement and no involvement		Informant/ codesigner		Informant		Codesigner	
		Q^a^	*P*	Q	*P*	Q	*P*
**Behavior (between categories)**							
	Narrative	0.05	.82	0.05	.82	0.03	.87
	Challenge	1.07	.30	1.07	.30	1.04	.31
	Characters	0.36	.55	1.80	.18	4.38	.04
	Game world	0.25	.62	0.25	.62	3.99	.046
	Language	0.79	.37	0.79	.37	0.10	.75
	Rewards	0.85	.36	0.85	.36	N/A^b^	
	Levels	N/A		N/A		N/A	
	Controls	0.37	.54	0.37	.54	N/A	
	Action/interactivity	0.11	.74	0.54	.47	N/A	
**Behavioral determinants (between categories)**							
	Narrative	0.43	.51	1.35	.24	0	.99
	Challenge	2.93	.09	N/A	N/A	11.23	.001
	Characters	1.17	.28	1.27	.26	0.21	.65
	Game world	0.32	.57	0.05	.83	0.59	.44
	Language	0.01	.93	0.46	.50	0.59	.44
	Rewards	2.07	.15	0.20	.66	N/A	
	Levels	7.02	.008	7.02	.008	N/A	
	Controls	N/A		N/A		N/A	
	Action/interactivity	0.48	.49	0.48	.49	N/A	

^a^Cochran’s Q test: homogeneity statistic (mixed effects).

^b^N/A: not applicable/not available due to insufficient observations.

PD in designing narratives, language, rewards, controls, and actions or interactivity did not relate significantly to game study effectiveness on behavior or its determinants. Significant differences were found for PD for creating the challenge, levels, character looks, and game world design. Involving users in PD on the challenge and levels related to higher game study effectiveness, whereas involvement in PD in character looks and game world design was associated with lower game study effectiveness. These differences in effects are discussed more in detail below.

When creating the challenge, there was a significant difference when considering codesign separately. Game studies where the challenge was codesigned with users (g=0.791, 95% CI 0.447 to 1.135, *P*<.001, k=4, n=318) were significantly more effective (Q=11.23, *P*=.001) in changing behavioral determinants than those where users had not been involved in codesign (g=0.192, 95% CI 0.128 to 0.257, *P*<.001, k=12, n=5837).

A significant relationship was also noted between user involvement as either informant or codesigner and game study effectiveness on behavioral determinants. Game studies where users were involved in the creation of levels as either informants or codesigners (g=0.771, 95% CI 0.347 to 1.196, *P*<.001, k=3, n=231) were significantly more effective (Q=7.02, *P*=.008) than game studies where users were not involved as informants or codesigners in level design (g=0.191, 95% CI 0.130 to 0.253, *P*<.001, k=13, n=5294). The same significant finding was noted when analyzing user involvement as informants separately, but could not be assessed for codesigners separately.

User involvement as codesigner, in the creation of characters, related to a significant difference in game study effectiveness on behavior. Game studies where users were involved in codesign of the characters (g=-0.022, 95% CI -0.128 to 0.083, *P*=.68, k=4, n=1485) were significantly less effective (Q=4.38, *P*=.04) at changing behavior than game studies where users were not involved in codesign (g=0.107, 95% CI 0.047 to 0.168, *P*<.001, k=10, n=6531). Differences in game study effectiveness on behavior were not significant when users were involved only as informants (Q=1.70, *P*=.18). Similarly, there was a significant difference in game effectiveness on behavior when only considering codesign.

Game studies where users had been involved in codesign of the game world (g=-0.075, 95% CI -0.232 to 0.082, *P*=.35, k=1, n=624) were significantly less effective (Q=3.99, *P*=.046) at changing behavior than game studies where users had not been involved in codesign (g=0.095, 95% CI 0.039 to 0.150, *P*=.001, k=13, n=7392). Note that this finding is based on only one study, albeit with a sufficient sample size, where codesign had been applied in creating the game world. Differences in game study effectiveness on behavior were not statistically significant when users were involved only as informants (Q=0.05, *P*=.83).

##### Educational Content

There were no significant differences (Q=0.01, *P*=.94) between the effectiveness of game studies on behavior when users were involved in PD of the educational content (g=0.084, 95% CI 0.030 to 0.144, *P*=.003, k=11, n=7027) or where users were not involved (g=0.078, 95% CI -0.144 to 0.301, *P*=.49, k=3, n=989). There were also no significant differences (Q=1.56, *P*=.21) between the effectiveness on behavioral determinants where users were (g=0.196, 95% CI 0.113 to 0.278, *P*<.001, k=11, n=4269) or were not involved in PD of educational content (g=0.381, 95% CI 0.102 to 0.659, *P*=.007, k=5, n=1886). Similar results were noted when analyzing the relationship between PD in educational content with effectiveness, separately for involvement as informant or as codesigner (see Multimedia Appendix 3, Table A3-6).

## Discussion

### Principal Findings

This study assessed whether user involvement PD in serious games for healthy lifestyle promotion related to game study effectiveness, and if this relationship varied by target group and study characteristics, by the design role and areas in which users were active participants. To our knowledge, this is the fırst study that conducted a meta-analysis of the relationship of user involvement and PD with serious game effectiveness.

More than half of the game studies involved users to some extent, with 25 out of 61 (41%) game studies having involved users as either informants or codesigners. User involvement in early stages of game design was previously reported to be low [72]; we did not find this in our study. However, this discrepancy may be influenced by the large number of recent game studies included in our study, since user involvement in serious game design was suggested to be on the rise recently [72].

Our first research question examined whether user involvement related significantly to game study effectiveness. Findings showed that serious game studies where the target group was involved in PD (ie, as informants or as codesigners) were, surprisingly, less effective in changing health behavior than when the target group was only involved as *testers* in the game. This was especially so for recent game studies and for game studies evaluated among an, on average, adult audience. Games developed with PD were also less effective at increasing self-efficacy (ie, how competent users perceive themselves to be in adopting a healthy lifestyle) than games developed without user involvement, in recent game studies and in studies evaluated among an, on average, younger audience. These results deviate from conclusions from a systematic review on nongame interventions, where higher community participation in the design process was associated with a higher achievement of health outcomes [73]. These conclusions, however, were obtained from *vote counting* (ie, correlation between number of outcomes achieved and degree of community engagement) and were not meta-analytic findings, which would have taken sample sizes and strength of effects across studies into account.

Several hypotheses can be advanced to explain these unexpected findings. First, users may need to be more than just a member of the target group; for example, they need sufficient subject-domain or design expertise to create a successful partnership with game designers [74]. This expertise may help avoid the risk of users focusing on irrelevant aspects, or on ideas that conflict with the pedagogical goals of the game [28,36]. Information on expertise level could be surmised for only two studies, where users were full members of the design team. Future teams intending to build a serious game with PD may test selecting users with content or design expertise, adjusting for lack of expertise, for example, by choosing appropriate techniques [75], or taking time to create user expertise [28,75]. Lack of experience with PD on the professional side, for example, not providing clear instructions or expectations, insufficient positive reinforcement, and not succeeding in creating a shared goal, could also lower the success of PD [76,77], which may have been operating in the studies included in this meta-analysis. Studies using PD in a school context noticed that without clear guidance, instructions, or encouragement from educators, children only did the minimum required, did not use all functionalities, and were reluctant to experiment and revise. When users did the bare minimum, they seemed to not know or share the overarching goal of the design process [76,77]. These findings also applied to adults where, additionally, not feeling recognized for their intellectual contribution could hinder the success of PD [77]. Information on these issues was not available for this review.

As a second hypothesis, codesign techniques (eg, storyboarding and paper prototyping) may need to be adjusted to the level of user design experience, users’ cognitive abilities, and stage of game development [74,75]. This was not described in most included studies, but may be associated with the quality of idea generation [75], and may overcome several barriers influencing codesign, such as users tending to think along familiar lines instead of exploring new avenues, or forgetting or being afraid to bring up ideas when generating ideas in a group [75]. Future serious game studies using PD should more clearly describe the process and techniques used [22], to further advance our understanding of how PD may relate to game effectiveness.

Some age differences were noted. Although PD related to lower effectiveness in both age groups, this lower effectiveness was shown in different outcomes. PD related to a lower effectiveness on self-efficacy for a younger audience, and a lower effectiveness on behavior for an, on average, adult audience. While informant design and codesign are classifications that apply both to PD with children and adults [75], the specific techniques used within these approaches (eg, comic boarding and sticky notes) may not be equally effective among children and adults. It could be hypothesized that younger participants may be tempted to add game features that enhanced the game experience and enjoyment. However, these may lead to an increased cognitive load, ultimately decreasing learning outcomes [78] and perceived competence in performing the target behavior. Adults involved in PD may not have been aware of a gap between positive behavioral intentions and behavioral performance and the methods needed to bridge this gap, such as goal-setting techniques [79]. Further research and possibly adjustment of techniques by age group are therefore warranted. More detailed descriptions of the process and techniques used in PD may also aid in understanding the lower effectiveness of PD in recent game studies. It seems from Table 2 that older game studies used more informant design, whereas newer studies used more codesign. These codesign methods may not yet be as well established as informant design methods or, given a recent proliferation of PD during game development [72], may not be adequately applied or used after insufficient training in PD. These are hypotheses that remain to be tested in future research.

Third, since our findings showed significant effectiveness differences by forms of PD, further examination of differences between roles of informants and codesigners might provide an explanation for differences in effectiveness. Case studies have suggested involving users as informants is more beneficial than involving them as codesigners [80,81], since users are often unable to consider game characteristics in relation to the learning objectives [81]. Our meta-analysis showed that the number of design elements where users were involved as informant or as codesigner did not significantly relate to game effectiveness on behavior or behavioral determinants.

Differences were found between involvement as informant or as codesigner in dynamics and game effectiveness on behavioral determinants. When examining user roles separately, a positive association was only significant when users were involved as informants and not when they were codesigners. This confirms observations from case studies that involving users as informants may be more effective than as codesigners in certain design elements [80,81].

User involvement as codesigner related to higher game effectiveness when involved in creating the game challenge, but to lower effectiveness if users were involved as codesigners in creating characters or in designing the looks of the game world. Different methods in PD, such as Informant Design [80], Bonded Design [75], and Co-design or Cooperative Inquiry [74], vary in the roles participants take in the process [75]. In Informant Design, users are asked to provide input and feedback at some, but not all, development stages. They provide suggestions, but do not carry out the design itself. In both Bonded Design and Cooperative Inquiry, users are involved in the creation of the material itself, and are considered integral design partners. In Bonded Design, user involvement may span a shorter time or may be restricted to certain development stages, whereas in Cooperative Inquiry, users are partners throughout the whole development process [75]. Our findings seem to provide more support for Informant Design than for Bonded Design (ie, shorter periods as codesigner) or Cooperative Inquiry (ie, continuous codesigning). Involving the target group as codesigners requires a lengthy iterative process compared to Informant Design, and compared to only pilot-testing a finalized version [28]. Possibly, there was insufficient time available in serious game design projects to properly execute codesign. Only 6 games using PD in our meta-analysis involved users throughout the entire design process, whereas 7 studies involved users in only one design element. This may indicate that most studies using codesign partners opted for Bonded Design rather than full and continuous cooperation.

Lastly, when users were involved in design decisions on game dynamics, specifically on creating levels, or were involved in creating the challenge, effectiveness at changing behavioral determinants was higher. Most game studies, however, involved users in the aesthetic parts of the design, such as character, narrative, or game world creation. These did not relate to game study effectiveness, or even related negatively to effectiveness. The positive relationship of PD in creating the game challenge, game levels, and dynamics with game study effectiveness may derive from the fact that these elements are more important to game study effectiveness in general. Involving users in trivial aspects not associated with effectiveness may be counterproductive and difficult to defend from a cost-effectiveness point of view.

The importance of user involvement in game dynamics may also be explained by the need to adjust the game demands to the cognitive and technical abilities of the users, since PD may help in creating better tools by assessing the cognitive processes of how users interact with technology [82].

In summary, our findings demonstrated that informant roles may have a stronger link to effectiveness than codesign, depending on the design element. Users should be involved in decisions that relate to the game dynamics (eg, levels) and to the challenge (eg, game type). Although PD in serious games for healthy lifestyle promotion was associated with lower effectiveness than only pilot-testing a version developed by professionals, several recommendations were made to increase effectiveness of PD in serious game design. Since other health interventions indicated that early user involvement was important to achieve reach, adoption, and sustained implementation of the intervention [83], it is essential to more fully understand the role of PD in serious games and how to optimize it, as all these elements together—see, for example, the Reach, Effectiveness, Adoption, Implementation, and Maintenance (RE-AIM) framework—determine the public health impact of health-promoting interventions [84,85].

### Limitations and Directions for Future Research

Some limitations to this meta-analysis need to be noted. First, "no evidence for an effect" does not equal "evidence for no effect." In some areas, analyses were likely statistically underpowered or were not available due to insufficient observations (eg, codesign roles per design element). Second, moderator analyses are equivalent to correlational analyses. There is always the possibility of a hidden, third variable. For example, a lower effectiveness for game studies developed in codesign may be influenced by lack of time in the project for optimal execution of codesign, insufficient user expertise, and a mismatch between techniques and user roles, expertise, and design phase. A more rigorous description of PD processes and of the application of recommended methods and techniques is needed in future PD game research, which would also enable a uniform coding based on what is reported in papers. Third, publication bias on the effects for behavioral determinants was noted, as reported elsewhere [2]. The reported effect sizes for these outcomes may be overestimated as a result. Lastly, no information was available on the impact of the input provided by users on the eventual game. Given often-conflicting opinions and goals between users and professionals [23], it is possible users’ input was not integrated into final versions of the games included in the meta-analysis.

### Conclusions

Our findings indicate that serious game studies for healthy lifestyle promotion developed with PD were less effective than game studies where users only pilot-tested a version designed by professionals. However, significant differences existed between the effectiveness of game studies developed with PD, suggesting certain types of PD may be more effective. This was the case for game studies where users were involved as informants in the design of game dynamics (eg, game levels). Involving users as codesigners for character or game world creation was less effective than not involving users in codesign for these game features. User involvement in designing the game challenge as informant or codesigner, on the other hand, related to higher game study effectiveness. These findings suggest PD should be mostly focused on areas crucial for general game study effectiveness, such as game dynamics and the challenge, whereas involvement in more trivial aspects, such as other aesthetic components, may be counterproductive. Involving users in user testing and informant roles may be more beneficial than as codesigners. Further research is needed to more fully explore how to incorporate PD into serious game design.

## Multimedia Appendix 1

Coding frame.

## Multimedia Appendix 2

Research protocol.

## Multimedia Appendix 3

Tables on moderator analyses.

## Abbreviations

C: user involvement as codesigner
Ch: challenge
CINAHL: Cumulative Index to Nursing and Allied Health Literature
CIPER: Interdisciplinary Centre for the Study of Human Performance
CLV: character looks/voice
Co: controls
DiGRA: Digital Games Research Association
F: feedback
FWO: Research Foundation-Flanders
GL: game world looks
I: user involvement as informant
IA: interactivity/action
k: number of studies
L: levels
LC: learning content
LW: language, wording
MDA: Mechanics, Dynamics, Aesthetics
N: narrative
N/A: not applicable
PD: participatory design
PRISMA: Preferred Reporting Items for Systematic Reviews and Meta-Analyses
R: rewards
RE-AIM: Reach, Effectiveness, Adoption, Implementation, and Maintenance
T: tailoring
UCD: user-centered design
USDA/ARS: United States Department of Agriculture/Agricultural Research Service
