# Supplementary material for: Is Participatory Design Associated with the Effectiveness of Serious Digital Games for Healthy Lifestyle Promotion? A Meta-Analysis
Source: J Med Internet Res. 2016 Apr 29;18(4):e94. doi: 10.2196/jmir.4444 (PMC4867751; doi:10.2196/jmir.4444)
Supplement: Supplementary file 1 [file jmir_v18i4e94_app1.pdf]

Framework component

Role

Definition

Clarification on how this was used

Aesthetics

Narrative

Informants

Users were asked for their preferences, suggestions for a narrative

Co-design

Users were involved in writing the narrative, writing parts of dialogue

Challenge

Informants

Users were asked for their preferences, ideas about the type and level of challenge (e.g. quiz, mystery, detective, jump ‘n run, shooter)

Co-design

Users were involved in creating the challenge (decide which type of challenge, determine difficulty levels, etc.)

Character looks

Informants

Users were asked about preferences, suggestions, about what the characters should look like

Co-design

Users were involved in creating the characters (drawing, choosing style,…)

Look of game world

Informants

Users were asked about preferences, suggestions about where the game should take place, which colors to use, which background elements to include

Co-design

Users were involved in creating the game world (e.g. drawing the setting, deciding which background elements to include)

Language

Informants

Users were asked about jargon, typical phrases, how characters should speak

Co-design

Users were asked to write sentences, decide on words, phrases, language style to use

Dynamics

Rewards

Informants

Preferences or suggestions were elicited from users on rewards (scoring, badges,…)

Co-design

Users decided on and created the reward system (scoring, badges,…)

Personalization

Informants

Suggestions for which elements to include in personalization were elicited from users

Co-design

Users decided and created the types of personalization available (e.g. designed hats, hair style, …)

Levels

Informants

Users were asked about number of levels and how to level up / down

Co-design

Users chose how to divide the target challenge into several levels, and decided on the strategy of how to change from one level to another

Mechanics

Actions/ interactivity

Informants

Users were asked for their preferences and suggestions on the actions (clicking, running, jumping, …) possible in the game

Co-design

Users designed and decided on the actions

Controls

Informants

Users were asked about their preferences and suggestions for controls (mouse, keys, …)

Co-design

Users decided on and designed the controls to use

Learning content

Informants

Users were asked for their preferences, suggestions for which topics and elements are important related to the health behavior

Co-design

Users designed (some) of the educational content themselves, e.g. by choosing which behaviors to work on (e.g. protected sex but not abstinence, strategies to say no to undesired sexual behavior, adding recipes for health diet,..)

Learning / Behavior change strategies

Feedback

Informants

Users were asked for their preferences on form of feedback (immediate, text, visual, auditive,…) and content (positive, how much)

Co-design

Users designed the feedback themselves

Individual tailoring

Informants

Users were asked about important criteria for tailoring the content (challenge or feedback) and how to tailor

Co-design

Users designed the tailored content

Other strategies

Informants

Describe any other strategy that may qualify as a learning or behavioral change strategy here (not already covered under game aspects above)

Co-design

Please specify

Other characteristics

Definition

Clarification on how this was used

Type of target group

Who was involved?

1. Only stakeholders

2. Only end users

3. End users and stakeholders (e.g. teachers, family, health professionals)

Number of consultations

With end users

Code the specific number of consultations reported, unless a group was established to guide the design at every step, which should be coded as 'constant'

With stakeholders

Code the specific number of consultations reported, unless a group was established to guide the design at every step, which should be coded as ‘constant’

Sampling strategy

Sampling method

1. Self-selected (volunteers), convenience sample
2. Purposive sampling
3. Snowball sampling
4. At random (e.g. from list)
5. Other

Similarity to population of end users

Were criteria used to ensure representativeness of the involved end users for the full population of end users?

1. No
2. Yes
3. Unsure

Please specify which

Similarity to population of stakeholders

Were criteria used to ensure representativeness of the involved end users for the full population of stakeholders?

1. No
2. Yes

3. Unsure

Please specify which

Sample size

User group

Total number of participants in the user group (adding up all different informants over several consultations. If one group guided the whole process, do not add these up over several consultations)

Stakeholders

Total number of participants in the user group (adding up all different informants over several consultations. If one group guided the whole process, do not add these up over several consultations)
