# Supplementary material for: Is Participatory Design Associated with the Effectiveness of Serious Digital Games for Healthy Lifestyle Promotion? A Meta-Analysis
Source: J Med Internet Res. 2016 Apr 29;18(4):e94. doi: 10.2196/jmir.4444 (PMC4867751; doi:10.2196/jmir.4444)
Supplement: Supplementary file 2 [file jmir_v18i4e94_app2.pdf]

1. The effectiveness of digital serious games to promote healthy lifestyles: a meta-analysis

1. Objective

To investigate the effectiveness of digital serious games as a tool to promote healthy lifestyle behavior (health responsibility, healthy diet, physical activity, stress management, interpersonal support, self-actualization) or its determinants.

2. Criteria for considering studies for this review

1. Type of studies

Only designs in which an effect size could be calculated on behavior or its determinants, based on an experimental or quasi-experimental design with at least 10 participants in each arm were included. Case studies, usability studies and studies only reporting effects on game enjoyment or motivation to play the game were hence excluded. Studies which reported the same outcomes from identical or overlapping samples in several articles, were only included once (most complete sample / most recent). If different outcomes were mentioned in several articles from identical or overlapping samples, these were grouped in one study.

2. Type of participants

Studies with participants from all ages and both genders were included. Studies could be conducted with either a general population or with patient groups, as long as the intervention type met the inclusion criteria (see 2.3).

3. Type of intervention

1. Healthy lifestyle promotion

Healthy lifestyles are modifiable determinants of a wide range of health issues and diseases, such as cancer, cardio-vascular diseases, stroke, dementia, mental health, and diabetes [1-4]. By increasing multiple healthy lifestyles, all-cause mortality can decrease with 66% in initially healthy adults [5]. Healthy lifestyle behaviors can furthermore increase quality of life in a general population [6] and attenuate the negative effects of illness on quality of life among chronic patients [7]. While health is also influenced by factors outside of the person's control [8] and adopting healthy lifestyles does not preclude the need for specialized care [9], the mental and physical health benefits of healthy lifestyles cause their promotion to be of great public health importance.

In this study, healthy lifestyles were defined based on the Health-promoting Lifestyles Profile scale, a validated and reliable measure of a multi-dimension health-promoting lifestyle, i.e. self-initiated behavior and attitudes that preserve or promote health and well-being [10]. The dimensions included were: Self-Actualization (e.g. feeling happy, having a purpose, setting goals for the future), Health Responsibility (e.g. having regular medical check-ups, attend prevention programs), Exercise (e.g. stretching, vigorous exercise), Nutrition (e.g. eating breakfast, having three meals per day), Interpersonal Support (e.g. maintain meaningful relationships, praise others), and Stress Management (e.g. relaxing, using stress control).

Some changes were made to these dimensions to closer fit with existing games. As games frequently targeted multiple health-promoting lifestyles, the dimensions 'Self-actualization' and 'stress management' were grouped under 'mental health promotion' to closer reflect serious games that have been developed. Nutrition and physical activity/exercise were also grouped since they frequently co-occur in games for health promotion. Health responsibility comprised both general health maintenance behavior and illness self-management. Hence categories that were used were: mental health promotion, social behavior, nutrition and physical activity, and health responsibility.

Games that were solely therapeutic in nature, which provided treatment that did not reflect self-initiated behavior to preserve or promote health and well-being, such as e.g. rehabilitation, treatment support, preparation for surgery, facilitating treatment decisions, were

not included. Games that aimed to increase skills to reach other goals than health, such as e.g. increasing athletic performance, were also not included.

Games that taught behavior in a therapeutic setting but aimed to result in self-initiated continuation of this behavior to promote health and well-being (e.g. psycho-education, illness self-management) were however included.

While variation existed in terms of the specific behavioral action required in these different health dimensions, the different types of health promoting behaviors have been meaningfully studied together in previous meta-analyses, e.g. on computer-delivered interventions for health promotion [11] on internet-delivered interventions for health promotion [12] and on e-health interventions targeted at children [13].

2. Digital serious games

A game was defined as organized play having a set of rules by which to play and a goal, which creates a challenge, provides feedback or shows outcomes, entails interaction and has a topic [14]. A serious game was defined as a game that had both educational goals and entertainment as a purpose [14,15]. Games only developed to entertain were hence not included. This excluded all games that were not tailor-made for educational purposes, e.g. Wii exergames. Although these games can have benefits on healthy lifestyles [16], tailor-made serious games provide additional benefits over what can be obtained with commercial games not developed to educate: they allow to provide disease-specific information, model positive health behaviors and provide opportunities to practice skills for healthy lifestyles [17].

Digital serious games used computerized platforms, such as CD-ROM, video consoles, computer, Internet, tablet PCs or smartphones. These digital interventions provided an opportunity to tailor. Tailored interventions are considered more effective to use in health behavior change than non-tailored interventions [11]. This definition of digital serious games excluded board games or face-to-face, physical games.

Games of any game intensity or play duration were accepted.

Studies were only included if they reported data that allowed the computation of an effect size for at least one of the following outcome measures of healthy lifestyle or its determinants, and if they randomly assigned individuals or known groups to an experimental and control condition.

For each study an effect size was calculated directly from means (or medians, event rates, odds ratios, F statistic for change, i.c. group*time) and SDs (or confidence intervals) with Hedges' formula for Hedges g, correcting for small sample sizes [18]. A negative Hedges g indicated that the serious game reduced adoption of a healthy lifestyle or its determinant compared to the control condition, a positive Hedges g indicated that the game increased the healthy lifestyle adoption or its determinant compared to the control condition. In cases where the intervention's effect targeted a reduction of unhealthy lifestyles (e.g. reduction in sedentary behavior), the computed sign of the effect size was reversed to ensure all positive differences indicated a greater improvement in healthy lifestyles for the treatment group compared to the control group. The model used was a random effects model given the variation between the studies in interventions, participants and measurement instruments.

Only studies that experimentally manipulated healthy lifestyles or their determinants were included. Studies that reported the health effects of spontaneous use of or exposure to games were excluded. These studies were correlational and cross-sectional.

Experimental and quasi-experimental studies may include:

- Experimental (i.c. randomly assigning individuals to control and intervention condition): Pre-test post-test control group design (RCT); post-test only control group design
    - o The effects of the game were compared between subjects in the intervention group and control group on post-measurement, taking measures on pretest into account

    - The effects of the game were compared between subjects in the intervention group and control group on post-measurement
- Quasi-experimental (i.c. randomly assigning individuals to control and intervention condition): non-equivalent groups pre-test post-test design; non-equivalent post-test only control group design
    - The effects of the game were compared between subjects in the intervention group and control group on post-measurement (taking measures on pretest into account)

Control conditions could consist of no intervention, of an intervention of a different type (e.g. website, leaflet, treatment as usual) or of a game of a different type (e.g. a non-tailor made game, game with no educational purposes, less immersive game). Control conditions that used the same game in content and layout but in a different intensity or game duration, were not accepted. Within subject studies (one group pretest-posttest) have no control condition and were not accepted. Sensitivity analyses were performed to assess whether the quality of the study impacted the outcomes.

For studies which used multiple dependent comparison groups within one study (e.g. several intervention groups and one control condition; or several control conditions and one intervention group), the multiple groups were combined using the following formula's [19]:

	Group 1	Group 2	Combined groups
Sample size	N_1	N_2	$N_1 + N_2$
Mean	M_1	M_2	$(N_1M_1 + N_2M_2) / (N_1+N_2)$
SD	SD_1	SD_2	$\sqrt{} \; (((N_1-1)SD_1^2 + (N_2-1)SD_2^2 +((N_1N_2)/(N_1+N_2)) (M_1^2+M_2^2-2M_1M_2)) / N_1+N_2 -1)$

If the study contained multiple independent comparisons (i.c. each intervention condition was compared to one and only one control condition), these were included as separate sub-studies (study name a, study name b). If a study contained multiple dependent comparisons and no average measures could be computed (e.g. F statistics, odds ratios were reported), one intervention group was chosen which closest reflected our research questions.

When the multiple dependent intervention groups differed on a characteristic of which we wished to assess the influence on the games' effectiveness (see moderator analyses), the following approach was applied:

1. The mean across several intervention groups (e.g. mean across stand-alone and multi-component version of one game) was used for all main analyses
2. In the moderator analyses, only the intervention group was included that belonged to the category with the fewest studies (e.g. only multi-component version)
3. When analyzing characteristics within one category of the moderator variable (e.g. analyzing stand-alone games and multi-component games separately), each intervention group was included separately.

3. Type of outcome measure

The primary outcome of the study needed to comprise healthy lifestyle behavior or its determinants (knowledge, attitudes, subjective norm, perceived barriers, self-efficacy, skills or behavioral intention). As a secondary outcome, symptoms or clinical effects could be included. Studies that only reported symptoms or clinical outcomes and no effects on behavior or behavioral determinants, were not included. In line with the main determinants that occur in most frequently used behavioral prediction models and which were considered the key determinants of behavior [20], we distinguished the following behavioral determinants:

- Behavior

- knowledge (as a prerequisite for other determinants)
- behavioral intention
- perceived barriers
- skills
- attitudes
- subjective norm
- self-efficacy

Each behavioral determinant and behavior was included separately as outcome. A separate meta-analysis was performed per outcome type, to avoid underestimating error when treating all outcomes as independent while they were not if they were derived from the same study and to avoid assigning more weight to studies that have measured more different outcomes. When several measurements were used within one study for one outcome type, the combined effect was computed as follows:

Combined effect size :

Combined variance of the mean :

Where Y_1, Y_2 stands for effect sizes, V_{Y1}, V_{Y2} stands for variances and r stands for correlation between Y_1 and Y_2. When r cannot be derived from the study reports, it is by standard set to 0.50.

If effects were only reported per subgroups and evaluated in independent samples (e.g. by gender), these were included as separate game evaluations (study name a, study name b). When two time points were provided, data from the shortest time point was included in the basic analysis. As can be expected that first measurement and follow-up measurement within one intervention would be correlated, the follow-up measurement was included in a separate meta-analysis on outcome comparisons at follow-up. When study characteristics differed between first evaluation and follow-up measurement (e.g. study design), they were included as separate game evaluations (study name a, study name b).

When values (e.g. means, odds ratios) were reported which were unadjusted for possible confounders (e.g. age), these were used by preference. If no unadjusted outcome values were available, the reported adjusted values were used in the calculation of the effect size. Sensitivity analyses comparing the effect size with and without studies with adjusted values have been conducted.

4. Moderator analyses

Moderator analysis was conducted to explain differences in effect sizes. For all moderator analyses, a mixed-effects model was used and Cochran's Q test and I^2 [21] were reported to investigate the degree of heterogeneity in effect sizes.

Moderator analyses were only conducted when there were at least 3 studies per category. If a category contained fewer than 3 studies, the moderator analyses was re-run without this category (when moderator variable contained >2 categories), unless the combined sample size consisted of at least 250 participants.

Meta-regression (methods-of-moments procedure) was performed for continuous moderators [22], where the slope (β) and its *p*–value indicated the importance of this moderator in understanding linear changes in effect sizes. To maintain the independence of the data, whenever necessary, effect sizes were averaged across different outcomes.

When a continuous moderator for one study contained several values (e.g. different follow-up durations across different outcomes), the rarest value on this moderator variable was used to provide maximum variation.

5. **Search methods for identification of studies**

Published studies were identified using electronic databases. Only papers and PhD dissertations that were published in English were selected. Reference and citation lists of papers were searched. Finally, a list of publications was presented to lead authors to ask for any other published papers.

Electronic databases searched were :

- CINAHL/EBSCO (1937- present)
- PUBMED (1966-present)
- PsychINFO (1887-present)
- Web of Science (1980- present)

All databases were searched using the terms

- Games OR
- Video games OR
- Interactive multimedia

AND health

The used terms were deliberately broad to be sensitive enough to also include studies dating back from before the term 'serious games' became popular.
Furthermore, the list of articles from the systematic search was completed with articles that were used in previous literature studies concerning serious games for health, that could also partly contain studies on healthy lifestyle promotion games:

- Baranowski, Buday, Thompson & Baranowski (2008)
- Connolly et al. (2012)
- DeShazo, Harris & Pratt (2010)
- Guse et al. (2012)
- Guy S, Ratzki-Leewing A, Gwadry-Sridhar F. (2011)
- Kharrazi, Lu, Gharghabi & Coleman (2012)
- Kato (2010)
- Papastergiou (2009)
- Primack et al. (2012)
- Rahmani & Austin Boren (2012)
- Shirong, Kharrazi, Gharghabi, Thompson (2013)

6. **Methods of the review**

   1. **Selection of the studies**

Initial screening based on title and abstract was performed by the first author (ADS). Full texts after this first selection were screened by two reviewers (ADS & WVL) for inclusion in the review. Reviewers were not blind for authors, institutions, journals and results.
If the article discussed a leisure game, a game that did not promote healthy lifestyles, that was not an original research paper, or that discussed effects on health from gaming where game was not used as an experimental condition, it was not included.
In addition to the abovementioned selection criteria, articles were only selected when the original authors classified the article as an article concerning serious games used for health promotion, by referring to games or video games and health promotion in the title, keywords or abstract.

Specific articles read in full-text were only included in the meta-analysis if the idea concerning serious gaming and health promotion was mentioned in the introduction section. Consensus was used to resolve disagreement regarding inclusion of the studies. If disagreement persists, a third reviewer (IDB) was consulted if necessary.

2. **Data extraction**

Data-extraction was conducted by one reviewer (ADS), after two authors (ADS & WVL) had conducted a pilot sample of 10 articles independently. This was performed by using a data extraction form specifically designed for this meta-analysis. If necessary, a third reviewer was brought in to resolve disagreements in the pilot sample. The coding categories were developed in an iterative process. The initial coding sheet was based on literature and distributed among the authors for their feedback. Next, the coding sheet was presented to leading serious game authors and developers at a local DiGRA meeting for their feedback, who suggested other important coding categories. This revised coding sheet was pilot tested by the reviewers on a sample of 10 articles and adjusted where necessary. The coding sheet was then finalized by the authors and used for double-coding on a third of the articles. Outcome data was also entered independently for a sample of 10 articles (ADS & SC).
Apart from this data-extraction, study characteristics such as sample and methodological quality were coded. To code study quality, the Quality Assessment tool for Quantitative Studies from the Effective Public Health Practice Project (EPHPP) (http://www.ephpp.ca/tools.html) was used. Quality was coded by one reviewer (ADS) after two authors (ADS & SC) had used this coding frame for a pilot sample of a third of included articles independently.

7. **Included variables**

Source characteristics

- Bibliographic reference
- Study ID (STIDxx)
- If a report presented two *independent studies* then a letter to the study ID number was added. For example, STIDxxa, STIDxxb,…
- Publication year
- Country of publication (1st author)
- Email address of corresponding author
- Dependent data ID (DDID xx)
  - o Studies often reported several outcome measures of a behavioral /determinant outcome using the same sample
  - o Studies sometimes compared several groups with a different type of game with the same control group.
  - o In all cases the calculated effect-sizes were not independent. The measure of dependent data index was increased by 1 for each added dependent measure. For example DDID:001, DDID:002, DDID:003

Experimental design

- Experimental:
  1. Pre-test post-test control group design
  2. Post-test only control group design
- Quasi-experimental:

3. Non-equivalent pretest-posttest control group design
4. Non-equivalent posttest only control group design

Sample characteristics

- Sample size control group
- Sample size serious game group
- Mean age; note: if the overall mean age was not reported and mean age of groups was reported, the overall mean was calculated ((M1*N1) +(M2*N2)) /(N1+N2)
- Proportion of females; note: if the overall proportion of females was not reported and proportion of females of both groups was reported, the overall mean was calculated ((M1*N1) +(M2*N2)) /(N1+N2)
- Health domain
  - Preventive behavior: included all forms of self-care, illness self-management, general preventive care and health responsibility (e.g. dental self-care, self-care in bacterial hygiene, sexual health, attending medical screening and having health check-ups)
  - Diet and physical activity: included all forms of healthy nutrition and sufficient physical activity and low sedentary behavior (e.g. eating three meals per day, stretching exercises, physical activity, sedentary behavior, weight status)
  - Mental health promotion: included what can promote positive mental health and avoid mental health problems and psychiatric disorders (e.g. self-esteem, look for opportunities for personal growth, stress management, depression or addiction prevention, promoting positive outlook on life, promoting cognitive functioning)
  - Social behavior: included positive human interactions, seeking social support, enhancing relationships with peers and family (e.g. social skills, not bullying)
  - The above categories were not mutually exclusive. For example it was possible that the intervention was aimed at illness self-management or preventive behavior in mental health (e.g. social skills for children with autism spectrum disorder, prevention of alcohol addiction). However to facilitate analyses, topics were be consistently grouped under one of these categories based on what similar included articles had presented most often as the primary focus of their study.

Reference List

1. Lopez AD, Mathers CD, Ezzati M, Jamison DT, Murray CJL: **Global and regional burden of disease and risk factors, 2001: systematic analysis of population health data.** *Lancet* 2006, **367:** 1747-1757.

2. Peel NM, McClure RJ, Bartlett HP: **Behavioral Determinants of Healthy Aging.** *Am J Prev Med* 2005, **28:** 298-304.

3. Fratiglioni L, Paillard-Borg S, Winblad B: **An active and socially integrated lifestyle in late life might protect against dementia.** *Lancet Neurol* 2004, **3:** 343-353.

4. Kim YS, Park YS, Allegrante JP, Marks R, Ok H, Cho KO *et al.*: **Relationship between physical activity and general mental health.** *Preventive Medicine* 2012, **55:** 458-463.

5. Loef M, Wallach H: **The combined effects of healthy lifestyle behaviors on all cause mortality: A systematic review and meta-analysis.** *Preventive Medicine* 2012, **55:** 163-170.

6. Bize R., Johnson JA, Plotnikoff RC: **Physical activity level and health-related quality of life in the general adult population: a systematic review.** *Preventive Medicine* 2007, **45:** 401-415.

7. Stuifbergen AK, Seraphine A, Roberts G: **An Explanatory Model of Health Promotion and Quality of Life in Chronic Disabling Conditions.** *Nursing Research* 2000, **49:** 122-129.

8. Dahlgren G, Whitehead M: *Policies and strategies to promote equity in health*. World Health Organization Regional Office for Europe; 1991.

9. von Korff M, Glasgow RE, Sharpe M: **ABC of psychological medicine. Organising care for chronic illness.** *BMJ* 2002, **325:** 92-94.

10. Walker SN, Sechrist KR, Pender NJ: **The Health-Promoting Lifestyle Profile: development and psychometric characteristics.** *Nursing Research* 1987, **36:** 76-81.

11. Portnoy DB, Scott-Sheldon LAJ, Johnson BT, Carey MP: **Computer-delivered Interventions for Health Promotion and Behavioral Risk Reduction: A Meta-Analysis of 75 Randomized Controlled Trials, 1988-2007.** *Preventive Medicine* 2008, **47:** 3-16.

12. Webb TL, Joseph J, Yardley L, Michie S: **Using the Internet to Promote Health Behavior Change: a Systematic Review and Meta-Analysis of the Impact of Theoretical Basis, Use of Behavior Change Techniques, and of Mode of Delivery on Efficacy.** *J Med Internet Res* 2010, **12:** e4-e29.

13. Cushing CC, Steele RG: **A Meta-Analytic Review of eHealth Interventions for Pediatric Health Promoting and Maintaining Behaviors.** *Journal of Pediatric Psychology* 2010, **35:** 937-949.

14. Prensky M: *Digital Game-Based Learning: Practical Ideas for the Application of Digital Game-Based Learning*, Edition 2007 edn. St. Paul, MN: Paragon House; 2007.

15. Ratan R, Ritterfeld U: **Classifying Serious Games.** In *Serious Games. Mechanisms and Effects*. Edited by Ritterfeld U, Cody M, Vorderer P. New York and London: Routledge; 2009:10-24.

16. Rahmani E, Boren SA: **Videogames and Health Improvement: A Literature Review of Randomized Controlled Trials.** *Games for Health Journal* 2012, **1:** 331-341.

17. Kato PM: **Video Games in Health Care: Closing the Gap.** *Review of General Psychology* 2010, **14:** 113-121.

18. Hedges L: **Distribution Theory for Glass's Estimator of Effect Size and Related Estimators.** *Journal of Educational Statistics* 1981, **6:** 128.

19. Higgings JPT, Green S: *Cochrane Handbook for Systematic Reviews of Interventions*, Version 5.1.0 edn. The Cochrane Collaboration; 2011.

20. Bartholomew LK, Parcel GS, Kok G, Gottlieb NH, Fernàndez ME: *Planning Health Promotion Programs. An Intervention Mapping Approach, 3rd Edition*. Jossey Bass; 2011.

21. Higgings JPT, Thompson SG, Deeks JJ, Altman DG: **Measuring inconsistency in meta-analyses.** *British Medical Journal* 2003, **327:** 557-560.

22. Thompson SG, Higgins JPT: **How should meta-regression analyses be undertaken and interpreted?** *Stat Med* 2002, **21:** 1559-1573.
