# Supplementary material for: Is Participatory Design Associated with the Effectiveness of Serious Digital Games for Healthy Lifestyle Promotion? A Meta-Analysis
Source: J Med Internet Res. 2016 Apr 29;18(4):e94. doi: 10.2196/jmir.4444 (PMC4867751; doi:10.2196/jmir.4444)
Supplement: Supplementary file 3 [file jmir_v18i4e94_app3.pdf]

Table A3-1. User involvement as moderator in game effectiveness (games published <2010).

Outcome	*n*	*k*	Hedges' g (95% CI)	P_g	*Q*	P_Q	I^2 Index
**BEHAVIOR**							
**TOTAL**[1]	6121	9	0. 102 [0.005; 0.199]	*.04*	310.05	*<.001*	97%
**User involvement (between categories)**	5989	8			1.15	*.28*	
**Within categories**							
No involvement	3036	2	0.728 [-0.460; 1.915]	*.23*	230.53	*<.001*	99%
Involvement only as tester	132	1	0.349 [0.007; 0.692]	*.046*	NA		
Involvement as informant or co-designer	2953	6	0.076 [-0.026; 0.177]	*.15*	8.87	*.16*	36%
**BEHAVIORAL DETERMINANTS**							
**TOTAL**[1]	6797	21	0.289 [0.197; 0.381]	*<.001*	52.17	*<.001*	62%
**User involvement (between categories)**	6797	21			1.41	*.49*	
**Within categories**							
No involvement	3718	7	0.278 [0.083; 0.473]	*.01*	29.96	*<.001*	80%
Involvement only as tester	558	5	0.443 [0.171; 0.715]	*.001*	9.05	*.06*	56%
Involvement as informant or co-designer	2521	9	0.266 [0.153; 0.379]	*<.001*	11.21	*.19*	29%
**SELF-EFFICACY**							
**TOTAL**[1]	2932	10	0.322 [0.236; 0.407]	*<.001*	18.28	*.03*	51%
**User involvement (between categories)**	2793	9			2.63	*.11*	
**Within categories**							
No involvement	1324	4	0.401 [0.292; 0.509]	*<.001*	1.46	*.69*	0%
Involvement only as tester	139	1	-0.052 [-0.382; 0.279]	*.76*	NA		
Involvement as informant or co-designer	1419	5	0.245 [0.093; 0.398]	*.002*	7.97	*.09*	50%
**CLINICAL OUTCOMES**							
**TOTAL**[1]	894	6	-0. 060 [-0.163; 0.168]	*.97*	5.17	*.40*	3%
**User involvement (between categories)**	835	5			6.34	*.43*	
**Within categories**							
No involvement	59	1	-0.115 [-0.620; 0.389]	*.65*	NA		
Involvement only as tester	269	2	0.083 [-0.156; 0.322]	*.50*	0.05	*.82*	0%

Involvement as informant or co-designer	566	3	-0.060 [-0.317; 0.198]	.*65*	4.34	.*11*	54%

Table A3-2. User involvement as moderator in game effectiveness (games published 2010-2014).

Outcome	*n*	*k*	Hedges' g (95% CI)	P_g	*Q*	P_Q	I^2 Index
**BEHAVIOR**							
**TOTAL**[1]	5563	13	0. 092 [0.019; 0.164]	*.01*	37.85	<*.001*	68%
**User involvement (between categories)**	5361	11			3.87	*.049*	
**Within categories**							
No involvement	202	2	0.292 [0.012; 0.572]	*.04*	1.03	*.31*	3%
Involvement only as tester	298	3	0.577 [0.074; 1.081]	*.03*	8.31	.02	76%
Involvement as informant or co-designer	5063	8	0.066 [-0.010; 0.142]	*.09*	6.68	*.46*	0%
**BEHAVIORAL DETERMINANTS**							
**TOTAL**[1]	6203	23	0.332 [0.247; 0.416]	<*.001*	55.75	<*.001*	61%
**User involvement (between categories)**	6203	23			1.95	*.38*	
**Within categories**							
No involvement	931	9	0.322 [0.173; 0.472]	<*.001*	8.99	*.34*	11%
Involvement only as tester	777	3	0.410 [0.264; 0.556]	<*.001*	0.91	*.64*	0%
Involvement as informant or co-designer	4495	11	0.264 [0.120; 0.409]	<*.001*	37.01	<*.001*	73%
**SELF-EFFICACY**							
**TOTAL**[1]	3466	10	0.131 [0.064; 0.199]	<*.001*	21.14	*.01*	57%
**User involvement (between categories)**	3466	10			8.17	*.02*	
**Within categories**							
No involvement	221	4	0.281 [0.009; 0.553]	*.04*	0.63	*.89*	0%
Involvement only as tester	295	2	0.483 [0.253; 0.714]	<*.001*	0.04	*.85*	0%
Involvement as informant or co-designer	2950	4	0.098 [-0.041; 0.237]	*.17*	8.83	*.03*	66%
**CLINICAL OUTCOMES**							
**TOTAL**[1]	309	3	0. 060 [-0.175; 0.501]	*.62*	1.20	*.55*	0%
**User involvement (between categories)**	309	3			NA		
**Within categories**							

No involvement	60	1	0.052 [-0.479; 0.582]	*.85*	NA		
Involvement only as tester	115	1	-0.090 [-0.467; 0.288]	*.64*	NA		
Involvement as informant or co-designer	134	1	0.204 [-0.161; 0.568]	*.27*	NA		

Table A3-3. User involvement as moderator in game effectiveness (average age of participants <18 years).

Outcome	*n*	*k*	Hedges' g (95% CI)	P_g	*Q*	P_Q	I^2 Index
**BEHAVIOR**							
**TOTAL**[1]	10200	16	0. 108 [0.050; 0.166]	*<.001*	320.30	*<.001*	95%
**User involvement (between categories)**	10068	15			1.26	*.26*	
**Within categories**							
No involvement	3238	4	0.540 [-0.230; 1.311]	*.17*	234.29	*<.001*	99%
Involvement only as tester	132	1	0.349 [0.007; 0.692]	*.046*	NA		
Involvement as informant or co-designer	6830	11	0.098 [0.040; 0.157]	*<.001*	7.88	*.64*	0%
**BEHAVIORAL DETERMINANTS**							
**TOTAL**[1]	9555	25	0.312 [0.220; 0.403]	*<.001*	73.01	*<.001*	67%
**User involvement (between categories)**	9555	25			0.23	*.89*	
**Within categories**							
No involvement	4210	10	0.313 [0.183; 0.442]	*<.001*	22.44	*.01*	60%
Involvement only as tester	322	3	0.425 [-0.057; 0.906]	*.08*	7.37	*.03*	73%
Involvement as informant or co-designer	5023	12	0.302 [0.168; 0.437]	*<.001*	37.07	*<.001*	70%
**SELF-EFFICACY**							
**TOTAL**[1]	5388	12	0.240 [0.164; 0.317]	*<.001*	35.91	*<.001*	69%
**User involvement (between categories)**	5199	11			9.66	*.002*	
**Within categories**							
No involvement	1321	4	0.377 [0.268; 0.486]	*<.001*	1.10	*.78*	0%
Involvement only as tester	139	1	-0.052 [-0.382; 0.279]	*.76*	NA		
Involvement as informant or co-designer	3878	7	0.127 [0.014; 0.241]	*.03*	15.54	*.02*	61%
**CLINICAL OUTCOMES**							
**TOTAL**[1]	818	5	0. 013 [-0.172; 0.199]	*.89*	5.67	*.23*	29%
**User involvement (between categories)**	835	5			NA		

**Within categories**							
No involvement	0	0	NA		NA		
Involvement only as tester	118	1	0.052 [-0.308; 0.411]	*.78*	NA		
Involvement as informant or co-designer	700	4	-0.001 [-0.217; 0.216]	*.99*	5.63	*.13*	47%

Table A3-4. User involvement as moderator in game effectiveness (average age of participants ≥ 18 years)

Outcome	*n*	*k*	Hedges' g (95% CI)	P_g	*Q*	P_Q	I^2 Index
**BEHAVIOR**							
**TOTAL**[1]	1484	6	0. 067 [-0.094; 0.229]	*0.41*	35.78	*<.001*	86%
**User involvement (between categories)**	1484	6			4.39	*.04*	
**Within categories**							
No involvement	0	0	NA		NA		
Involvement only as tester	298	3	0.577 [0.074; 1.081]	.03	8.31	*.02*	76%
Involvement as informant or co-designer	1186	3	0.009 [-0.161; 0.179]	*0.92*	4.09	*.13*	51%
**BEHAVIORAL DETERMINANTS**							
**TOTAL**[1]	3248	16	0.328 [0.231; 0.424]	*<.001*	32.86	*.01*	54%
**User involvement (between categories)**	3248	16			3.15	*.21*	
**Within categories**							
No involvement	380	5	0.279 [-0.154; 0.712]	*.21*	15.17	*.004*	74%
Involvement only as tester	935	4	0.410 [0.277; 0.542]	*<.001*	0.91	*.82*	0%
Involvement as informant or co-designer	1933	7	0.231 [0.082; 0.379]	*.002*	11.55	*.07*	48%
**SELF-EFFICACY**							
**TOTAL**[1]	1001	7	0.407 [0.281; 0.532]	*<.001*	2.50	*.87*	0%
**User involvement (between categories)**	1001	7			0.92	*.63*	
**Within categories**							
No involvement	165	3	0.453 [0.140; 0.765]	*.004*	1.41	*.49*	0%
Involvement only as tester	295	2	0.483 [0.253; 0.714]	*<.001*	0.04	*.85*	0%
Involvement as informant or co-designer	541	2	0.351 [0.181; 0.521]	*<.001*	0.14	*.71*	0%
**CLINICAL OUTCOMES**							
**TOTAL**[1]	175	2	-0. 042 [-0.350; 0.265]	*.79*	0.18	*.67*	0%
**User involvement (between categories)**	175	2			NA		
**Within categories**							

No involvement	60	1	0.052 [-0.479; 0.582]	.85	NA		
Involvement only as tester	118	1	-0.090 [-0.467; 0.288]	.64	NA		
Involvement as informant or co-designer	0	0	NA		NA		

Table A3-5. Meta-regression results of user involvement as informant or co-designer on game study effectiveness in behavior and behavioral determinants

			INFORMANT			CO-DESIGNER		
	K	N	β	95% CI	*P*	β	95% CI	*P*
**BEHAVIOR**								
All areas	14	8016	-0.002	-0.032; 0.029	*.92*	-0.007	-0.041; 0.027	*.69*
Aesthetics	14	8016	-0.001	-0.038; 0.035	*.94*	-0.024	-0.065; 0.017	*.27*
Dynamics	14	8016	0.074	-0.040; 0.187	*.20*		NA	
Mechanics	14	8016	-0.064	-0.161; 0.033	*.20*	0.063	-0.064; 0.190	*.33*
**BEHAVIORAL DETERMINANTS**								
All areas	16	6155	0.034	-0.039; 0.108	*.36*	0.036	-0.015; 0.089	*.16*
Aesthetics	16	6155	0.025	-0.058; 0.108	*.56*	0.046	-0.027; 0.120	*.22*
Dynamics	16	6155	0.235	0.079; 0.329	***.003***	0.157	-0.115; 0.428	*.26*
Mechanics	16	6155	-0.078	-0.343; 0.188	*.57*		NA	

n=combined participant sample size; k=number of studies; β : meta-regression weight; CI= confidence Interval; NA: not available, due to insufficient observations or variation

Table A3-6. Moderator analysis for the role of PD in educational content on the game study effectiveness outcomes on behavior and behavioral determinants.

Outcome	*n*	*k*	Hedges' g (95% CI)	*P*	*Q*	*P*	*I*² Index
**BEHAVIOR**							
**Involvement as informant (between categories)**	8016	14			0.06	*.81*	
**Within categories**							
No	1164	4	0.104 [-0.072; 0.280]	*.25*	5.95	*.11*	50%
Yes	6352	10	0.081 [0.022; 0.140]	*.007*	8.60	*.48*	0%
**Involvement as co-designer (between categories)**	8016	14			1.04	*.31*	
**Within categories**							
No	7341	13	0.068 [0.008; 0.128]	*.03*	13.64	*.32*	12%
Yes	675	1	0.194 [-0.041; 0.429]	*.11*	NA		
**BEHAVIORAL DETERMINANTS**							
**Involvement as informant (between categories)**	6155	16			2.92	*.09*	
**Within categories**							
No	1948	7	0.410 [0.160; 0.660]	*.001*	23.76	*.001*	75%
Yes	4207	9	0.182 [0.106; 0.258]	*<.001*	10.36	*.24*	23%
**Involvement as co-designer (between categories)**	6155	16			NA		
**Within categories**							
No	6093	14	0.241 [0.143; 0.340]	*<.001*	35.75	*.001*	64%
Yes	62	2	0.561 [-0.072; 1.194]	*.08*	1.61	*.21*	38%

n=combined participants sample size; k=number of studies; Hedges' g (random effects); CI= confidence Interval; Q= homogeneity statistic (mixed effects); I²=Inconsistency, a second measure of heterogeneity; NA: not available

Table A3-7. Moderator analysis for the role of user involvement in creating **narratives** on the game effectiveness outcomes on behavior and behavioral determinants.

Outcome	*n*	*k*	Hedges' g (95% CI)	*P*	*Q*	*P*	*I²* Index
BEHAVIOR							
Involvement as either informant or co-designer	4113	8			0.05	*.82*	
**No**	2357	4	0.049 [-0.070; 0.169]	*.42*	4.91	*.18*	39%
**Yes**	1757	4	0.072 [-0.083; 0.226]	*.36*	5.30	*.15*	43%
Involvement as informant	4113	8			0.05	*.82*	
**No**	2357	4	0.049 [-0.070; 0.169]	*.42*	4.91	*.18*	39%
**Yes**	1757	4	0.072 [-0.083; 0.226]	*.36*	5.30	*.15*	43%
Involvement as co-designer	4113	8			0.03	*.87*	
**No**	2815	6	0.068 [-0.024; 0.159]	*.15*	6.14	*.29*	19%
**Yes**	1299	2	0.045 [-0.217; 0.307]	*.74*	3.49	*.06*	71%
BEHAVIORAL DETERMINANTS							
Involvement as either informant or co-designer	2808	10			0.43	*.51*	
**No**	1233	5	0.352 [0.071; 0.633]	*.01*	21.99	<*.001*	82%
**Yes**	1575	5	0.244 [0.083; 0.405]	*.003*	5.67	*.23*	29%
Involvement as informant	2808	10			1.35	*.24*	
**No**	1382	8	0.385 [0.141; 0.630]	*.002*	24.45	*.001*	71%
**Yes**	1426	2	0.209 [0.039; 0.378]	*.02*	2.42	*.12*	59%
Involvement as co-designer	2808	10			0.00	*.99*	
**No**	1738	6	0.304 [0.076; 0.532]	*.009*	25.75	<*.001*	80%
**Yes**	1070	4	0.303 [0.177; 0.428]	<*.001*	2.66	*.45*	0%

n=combined sample size; k=number of studies; Hedges' g (random effects); CI= confidence Interval; Q= homogeneity statistic (mixed effects); I²=Inconsistency, a second measure of heterogeneity; NA: not applicable

Table A3-8. Moderator analysis for the role of user involvement in design of the **challenge** on the game effectiveness outcomes on behavior and behavioral determinants.

Outcome	*n*	*k*	Hedges' g (95% CI)	*P*	*Q*	*P*	*I*² Index
BEHAVIOR							
Involvement as either informant or co-designer	8016	14			1.07	*.30*	
**No**	5394	10	0.052 [-0.027; 0.132]	*.20*	11.89	*.22*	24%
**Yes**	2622	4	0.113 [0.029; 0.198]	*.008*	1.54	*.67*	0%
Involvement as informant	8016	14			1.07	*.30*	
**No**	5394	10	0.052 [-0.027; 0.132]	*.20*	11.89	*.22*	24%
**Yes**	2622	4	0.113 [0.029; 0.198]	*.008*	1.54	*.67*	0%
Involvement as co-designer	8016	14			1.04	*.31*	
**No**	7341	13	0.068 [0.008; 0.128]	<*.05*	13.64	*.32*	12%
**Yes**	675	1	0.194 [-0.041; 0.429]	*.11*	NA		
BEHAVIORAL DETERMINANTS							
Involvement as either informant or co-designer	6155	16			2.93	*.09*	
**No**	5777	11	0.194 [0.126; 0.262]	<*.001*	14.28	*.16*	30%
**Yes**	377	5	0.579 [0.144; 1.013]	*.009*	9.49	*.05*	59%
Involvement as informant	6155	16			NA		
**No**	5926	14	0.202 [0.133; 0.271]	<*.001*	18.00	*.16*	28%
**Yes**	229	2	0.575 [-0.246; 1.396]	*.17*	7.50	*.006*	87%
Involvement as co-designer	6155	16			11.23	***.001***	
**No**	5837	12	0.192 [0.128; 0.257]	<*.001*	14.32	*.22*	23%
**Yes**	318	4	0.791 [0.447; 1.135]	<*.001*	3.65	*.30*	18%

n=combined sample size; k=number of studies; Hedges' g (random effects); CI= confidence Interval; Q= homogeneity statistic (mixed effects); I²=Inconsistency, a second measure of heterogeneity; NA: not applicable

Table A3-9. Moderator analysis for the role of user involvement in designing **character looks** on the game effectiveness outcomes on behavior and behavioral determinants.

Outcome	*n*	*k*	Hedges' g (95% CI)	*P*	*Q*	*P*	*I*² Index
BEHAVIOR							
Involvement as either informant or co-designer	8016	14			0.36	*.55*	
**No**	3382	4	0.050 [-0.050; 0.150]	*.33*	1.84	*.61*	0%
**Yes**	4634	10	0.089 [0.010; 0.167]	*.03*	12.47	*.19*	28%
Involvement as informant	8016	14			1.80	*.18*	
**No**	4012	6	0.030 [-0.055; 0.115]	*.49*	3.15	*.68*	0%
**Yes**	4004	8	0.113 [0.026; 0.199]	*.01*	9.72	*.21*	5%
Involvement as co-designer	8016	14			4.38	***.04***	
**No**	6531	10	0.107 [0.047; 0.168]	*<.001*	7.49	*.59*	0%
**Yes**	1485	4	-0.022 [-0.128; 0.083]	*.68*	2.81	*.42*	0%
BEHAVIORAL DETERMINANTS							
Involvement as either informant or co-designer	6155	16			1.17	*.28*	
**No**	3251	8	0.197 [0.080; 0.314]	*.001*	13.15	*.07*	47%
**Yes**	2904	8	0.308 [0.145; 0.470]	*<.001*	22.36	*.002*	69%
Involvement as informant	6155	16			1.27	*.26*	
**No**	3571	9	0.200 [0.098; 0.302]	*<.001*	13.59	*.09*	41%
**Yes**	2584	7	0.326 [0.132; 0.519]	*.001*	22.31	*.001*	73%
Involvement as co-designer	6155	16			0.21	*.65*	
**No**	4178	12	0.272 [0.135; 0.408]	*<.001*	35.79	*<.001*	69%
**Yes**	1977	4	0.233 [0.138; 0.327]	*<.001*	3.04	*.39*	1%

n=combined sample size; k=number of studies; Hedges' g (random effects); CI= confidence Interval; Q= homogeneity statistic (mixed effects); I²=Inconsistency, a second measure of heterogeneity; NA: not applicable

Table A3-10. Moderator analysis for the role of user involvement in designing **game world looks** on the game effectiveness outcomes on behavior and behavioral determinants.

Outcome	*n*	*k*	Hedges' g (95% CI)	P	*Q*	P	I^2 Index
BEHAVIOR							
Involvement as either informant or co-designer	8016	14			0.25	.62	
**No**	4668	9	0.093 [0.017; 0.168]	.02	6.42	.60	0%
**Yes**	3348	5	0.058[-0.055; 0.171]	.31	7.89	.096	49%
Involvement as informant	8016	14			0.25	.62	
**No**	4668	9	0.093 [0.017; 0.168]	.02	6.42	.60	0%
**Yes**	3348	5	0.058[-0.055; 0.171]	0.31	7.89	.096	49%
Involvement as co-designer	8016	14			3.99	**.046**	
**No**	7392	13	0.095 [0.039; 0.150]	.001	10.69	.56	0%
**Yes**	624	1	-0.075 [-0.232; 0.082]	.35	NA		
BEHAVIORAL DETERMINANTS							
Involvement as either informant or co-designer	6155	16			0.32	0.57	
**No**	4765	11	0.241 [0.118; 0.363]	<.001	33.74	<.001	70%
**Yes**	1390	5	0.288 [0.179; 0.397]	<.001	2.88	0.58	0%
Involvement as informant	6155	16			0.05	0.83	
**No**	4827	13	0.257 [0.135; 0.378]	<.001	37.8	<.001	68%
**Yes**	1328	3	0.275 [0.163; 0.386]	<.001	0.144	0.93	0%
Involvement as co-designer	6155	16			0.59	0.44	
**No**	5172	13	0.239 [0.129; 0.349]	<.001	34.05	<.01	65%
**Yes**	983	3	0.354 [0.082; 0.626]	.01	2.63	.27	24%

n=combined sample size; k=number of studies; Hedges' g (random effects); CI= confidence Interval; Q= homogeneity statistic (mixed effects); I^2=Inconsistency, a second measure of heterogeneity; NA: not applicable

Table A3-11. Moderator analysis for the role of user involvement in creating the **language in the game,** on the game effectiveness outcomes on behavior and behavioral determinants.

Outcome	*n*	*k*	Hedges' g (95% CI)	*P*	*Q*	*P*	*I*² Index
BEHAVIOR							
Involvement as either informant or co-designer	8016	14			0.79	*.37*	
**No**	4921	10	0.100 [0.028; 0.172]	*.007*	8.11	*.52*	0%
**Yes**	3095	4	0.038 [-0.077; 0.154]	*.52*	5.66	*.13*	47%
Involvement as informant	8016	14			0.79	*.37*	
**No**	4921	10	0.100 [0.028; 0.172]	*.007*	8.11	*.52*	0%
**Yes**	3095	4	0.038 [-0.077; 0.154]	*.52*	5.66	*.13*	47%
Involvement as co-designer	8016	14			0.10	*.75*	
**No**	6717	12	0.089 [0.032; 0.146]	*.002*	9.95	*.54*	0%
**Yes**	1299	2	0.045 [-0.217; 0.307]	*.74*	3.49	*.06*	71%
BEHAVIORAL DETERMINANTS							
Involvement as either informant or co-designer	6155	16			0.01	*.93*	
**No**	4865	12	0.259 [0.141; 0.377]	*<.001*	32.28	*.001*	66%
**Yes**	1290	4	0.247 [0.020; 0.475]	*.03*	6.40	*.094*	53%
Involvement as informant	6155	16			0.46	*.50*	
**No**	4927	14	0.273 [0.156; 0.390]	*<.001*	35.87	*.001*	64%
**Yes**	1228	2	0.185 [-0.045; 0.414]	*.12*	3.22	*.07*	69%
Involvement as co-designer	6155	16			0.59	*.44*	
**No**	5172	13	0.239 [0.129; 0.349]	*<.001*	34.05	*.001*	65%
**Yes**	983	3	0.354 [0.082; 0.626]	*.01*	2.63	*.27*	24%

n=combined sample size; k=number of studies; Hedges' g (random effects); CI= confidence Interval; Q= homogeneity statistic (mixed effects); I²=Inconsistency, a second measure of heterogeneity; NA: not applicable

Table A3-12. Moderator analysis for the role of user involvement in design of **rewards** on the game effectiveness outcomes on behavior and behavioral determinants.

Outcome	*n*	*k*	Hedges' g (95% CI)	*P*	*Q*	*P*	I^2 Index
BEHAVIOR							
Involvement as either informant or co-designer	8016	14			0.85	*.36*	
**No**	5606	10	0.056 [-0.023; 0.134]	*.17*	11.07	*.27*	19%
**Yes**	2410	4	0.109 [0.028; 0.190]	*.008*	2.51	*.47*	0%
Involvement as informant	8016	14			0.85	*.36*	
**No**	5606	10	0.056 [-0.023; 0.134]	*.17*	11.07	*.27*	19%
**Yes**	2410	4	0.109 [0.028; 0.190]	*.008*	2.51	*.47*	0%
Involvement as co-designer	8016	14			NA		
**No**	8016	14	0.075 [0.017; 0.133]	*.01*	14.67	*.33*	11%
**Yes**	0	0					
BEHAVIORAL DETERMINANTS							
Involvement as either informant or co-designer	6155	16			2.07	*.15*	
**No**	5253	11	0.194 [0.120; 0.267]	*<.001*	13.95	*.18*	28%
**Yes**	902	5	0.464 [0.103; 0.824]	*.01*	20.00	*<.001*	80%
Involvement as informant	6155	16			0.20	*.66*	
**No**	5315	13	0.204 [0.126; 0.282]	*<.001*	17.62	*.13*	32%
**Yes**	840	3	0.428 [-0.023; 0.880]	*.06*	17.75	*<.001*	89%
Involvement as co-designer	6155	16			NA		
**No**	6093	14	0.241 [0.143; 0.340]	*<.001*	35.75	*.001*	64%
**Yes**	62	2	0.561 [-0.072; 1.194]	*.08*	1.61	*.21*	38%

n=combined sample size; k=number of studies; Hedges' g (random effects); CI= confidence Interval; Q= homogeneity statistic (mixed effects); I^2=Inconsistency, a second measure of heterogeneity; NA: not applicable

Table A3-13. Moderator analysis for the role of user involvement in design of **levels** on the game effectiveness outcomes on behavior and behavioral determinants.

Outcome	*n*	*k*	Hedges' g (95% CI)	*P*	*Q*	*P*	*I*² Index
BEHAVIOR							
Involvement as either informant or co-designer	8016	14			NA		
**No**	7882	13	0.070 [0.012; 0.128]	*.02*	13.52	.*33*	11%
**Yes**	134	1	0.274 [-0.092; 0.639]	.*14*	NA		
Involvement as informant	8016	14			NA		
**No**	7882	13	0.070 [0.012; 0.128]	*.02*	13.52	.*33*	11%
**Yes**	134	1	0.274 [-0.092; 0.639]	.*14*	NA		
Involvement as co-designer	8016	14			NA		
**No**	8016	14	0.075 [0.017; 0.133]	*.01*	14.67	.*33*	11%
**Yes**	0	0					
BEHAVIORAL DETERMINANTS							
Involvement as either informant or co-designer	6155	16			7.02	***.008***	
**No**	5294	13	0.191 [0.130; 0.253]	<*.001*	14.40	*.28*	17%
**Yes**	231	3	0.771 [0.347; 1.196]	<*.001*	3.51	*.17*	43%
Involvement as informant	6155	16			7.02	***.008***	
**No**	5294	13	0.191 [0.130; 0.253]	<*.001*	14.40	*.28*	17%
**Yes**	231	3	0.771 [0.347; 1.196]	<*.001*	3.51	*.17*	43%
Involvement as co-designer	6155	16			NA		
**No**	6093	14	0.241 [0.143; 0.340]	<*.001*	35.75	*.001*	64%
**Yes**	62	2	0.561 [-0.072; 1.194]	*.08*	1.61	*.21*	38%

n=combined sample size; k=number of studies; Hedges' g (random effects); CI= confidence Interval; Q= homogeneity statistic (mixed effects); I²=Inconsistency, a second measure of heterogeneity; NA: not applicable

Table A3-14. Moderator analysis for the role of user involvement in design of **controls** on the game effectiveness outcomes on behavior and behavioral determinants.

Outcome	*n*	*k*	Hedges' g (95% CI)	*P*	*Q*	*P*	I^2 Index
BEHAVIOR							
Involvement as either informant or co-designer	8016	14			0.37	*.54*	
**No**	7017	12	0.069 [0.003; 0.134]	*.04*	13.62	*.26*	19%
**Yes**	999	2	0.124 [-0.043; 0.290]	*.15*	0.692	*.41*	0%
Involvement as informant	8016	14			0.37	*.54*	
**No**	7017	12	0.069 [0.003; 0.134]	*.04*	13.62	*.26*	19%
**Yes**	999	2	0.124 [-0.043; 0.290]	*.15*	0.692	*.41*	0%
Involvement as co-designer	8016	14			NA		
**No**	7341	13	0.068 [0.008; 0.128]	*.03*	13.64	*.32*	12%
**Yes**	675	1	0.194 [-0.041; 0.429]	*.11*	NA		
BEHAVIORAL DETERMINANTS							
Involvement as either informant or co-designer	6155	16			NA		
**No**	6068	15	0.253 [0.153; 0.353]	<*.001*	39.12	<*.001*	64%
**Yes**	87	1	0.463 [-1.473; 2.399]	*.64*	NA		
Involvement as informant	6155	16			NA		
**No**	6068	15	0.253 [0.153; 0.353]	<*.001*	39.12	<*.001*	64%
**Yes**	87	1	0.463 [-1.473; 2.399]	*.64*	NA		
Involvement as co-designer	6155	16			NA		
**No**	6155	16	0.253 [0.154; 0.351]	<*.001*	39.19	*.001*	62%
**Yes**	0	0			NA		

n=combined sample size; k=number of studies; Hedges' g (random effects); CI= confidence Interval; Q= homogeneity statistic (mixed effects); I^2=Inconsistency, a second measure of heterogeneity; NA: not applicable

Table A3-15. Moderator analysis for the role of user involvement in design of **action and interactivity** on the game effectiveness outcomes on behavior and behavioral determinants.

Outcome	*n*	*k*	Hedges' g (95% CI)	*P*	*Q*	*P*	I^2 Index
BEHAVIOR							
Involvement as either informant or co-designer	8016	14			0.11	*.74*	
**No**	5634	9	0.087 [0.023; 0.152]	*.008*	6.03	*.64*	0%
**Yes**	2382	5	0.063 [-0.069; 0.194]	*.35*	8.29	*.08*	52%
Involvement as informant	8016	14			0.54	*.47*	
**No**	6309	10	0.095 [0.032; 0.157]	*.003*	6.76	*.66*	0%
**Yes**	1707	4	0.035 [-0.112; 0.182]	*.64*	6.70	*.08*	55%
Involvement as co-designer	8016	14			NA		
**No**	7341	13	0.068 [0.008; 0.128]	*.03*	13.64	*.32*	12%
**Yes**	675	1	0.194 [-0.041; 0.429]	*.11*	NA		
BEHAVIORAL DETERMINANTS							
Involvement as either informant or co-designer	6155	16			0.48	*.49*	
**No**	4388	12	0.283 [0.150; 0.461]	<*.001*	35.70	<*.001*	69%
**Yes**	1767	4	0.223 [0.119; 0.328]	<*.001*	3.29	*.35*	9%
Involvement as informant	6155	16			0.48	*0.49*	
**No**	4388	12	0.283 [0.150; 0.461]	<*.001*	35.70	<*.001*	69%
**Yes**	1767	4	0.223 [0.119; 0.328]	<*.001*	3.29	*0.35*	9%
Involvement as co-designer	6155	16			NA		
**No**	6155	16	0.253 [0.154; 0.351]	<*.001*	39.19	*.001*	62%
**Yes**	0	0			NA		

n=combined sample size; k=number of studies; Hedges' g (random effects); CI= confidence Interval; Q= homogeneity statistic (mixed effects); I²=Inconsistency, a second measure of heterogeneity; NA: not applicable
